# Insights into the Pseudocapacitive Behavior of Sulfurized Polymer Electrodes for Li–S Batteries

**DOI:** 10.1002/advs.202206901

**Published:** 2023-03-30

**Authors:** Nawraj Sapkota, Shailendra Chiluwal, Prakash Parajuli, Alan Rowland, Ramakrishna Podila

**Affiliations:** ^1^ Department of Physics and Astronomy Clemson University Clemson SC 29634 USA

**Keywords:** batteries, electrochemistry, lithium sulfur, pseudocapacitors, sulfurized polymers

## Abstract

Practical applications of sulfurized polymer (SP) materials in Li–S batteries (LSBs) are often written off due to their low S content (≈35 wt%). Unlike conventional S_8_/C composite cathodes, SP materials are shown to function as pseudocapacitors with an active carbon backbone using a comprehensive array of tools including in situ Raman and electrochemical impedance spectroscopy. Critical metric analysis of LSBs containing SP materials with an active carbon skeleton shows that SP cathodes with 35 wt% S are suitable for 350 Wh kg^−1^ target at the cell level if S loading >5 mg cm^−2^, electrolyte‐to‐sulfur ratio <2 µL mg^−1^, and negative‐to‐positive ratio <5 can be achieved. Although 3D current collectors can enable such high loadings, they often add excess mass decreasing the total capacity. An “active” carbon nanotube bucky sandwich current collector developed here offsets its excess weight by contributing to the electric double layer capacity. SP cathodes (35 wt% S) with ≈5.5 mg cm^−2^ of S loading (≈15.8 mg cm^−2^ of SP loading) yield a sulfur‐level gravimetric capacity ≈1360 mAh g_s_^−1^ (≈690 mAh g_s_^−1^), electrode level capacity 200 mAh g_electrode_^−1^ (100 mAh g_electrode_^−1^), and areal capacity ≈7.8 mAh cm^−2^ (≈4.0 mAh cm^−2^) at 0.1C (1C) rate for ≈100 cycles at E/S ratio = 7 µL mg^−1^.

## Graphical abstract

Beyond elemental sulfur electrodes for Li—S batteries, many researchers propose sulfurized polymers (SP) for circumventing polysulfide formation and achieving long cycling stability. The practical application of SP electrodes is often written off due to low S loading. It is shown that N doping in SP electrodes removes quantum capacitance limitations allowing them to function as pseudocapacitors with increased total capacity.

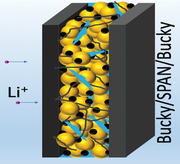

## 1 Introduction

Lithium–sulfur batteries (LSBs) have the potential for >400 miles driving range with practical capacities up to 500 Wh kg^−1^ (twice that of Li‐ion batteries or LIBs) at the pack level.^[^ ^1^, ^2^, ^3^ ^]^ In conventional LSBs, the cathode consists of insulating sulfur embedded into a conducting host, while a thin Li metal strip serves as the anode with a highly reversible reaction (S_8_ + 16 Li ↔ 8 Li_2_S), resulting in ≈2.15 V versus Li/Li^+^. The high specific capacity of sulfur (1675 mAh g^−1^) gives a theoretical energy density of 2500 Wh kg^−1^ for an LSB cell, an order of magnitude higher than that of LIBs. A major challenge with elemental sulfur is its octagonal form, which undergoes a series of structural and morphological changes during a charge–discharge cycle leading to the formation of soluble lithium polysulfides or LiPS (Li_2_S*_x_*, where 8 ≤ *x* ≤ 3) and insoluble LiPS (Li_2_S_2_/Li_2_S) in the electrolyte inside the porous cathode.^[^ ^4^, ^5^, ^6^, ^7^, ^8^ ^]^ Soluble LiPS intermediates eventually diffuse from the porous cathode into the electrolyte in the separator leading to severe active material (AM) loss and the so‐called “shuttle effect.”^[^ ^1^, ^2^, ^3^, ^9^, ^10^, ^11^, ^12^ ^]^ Diffused LiPS are preferentially oxidized to sulfur and redeposited near the top surface of the conductive cathode. This results in accumulation of sulfur‐species on the top surface of the cathode after repeated cycles resulting in loss of electric contact, blockage of ion transport into the cathode, increased electrode resistance, deactivated internal active materials, and fast cell failure. Sulfur/carbon (S/C) composites have been suggested as a strategy to tackle these material challenges,^[^ ^1^, ^2^, ^3^, ^5^, ^6^, ^8^, ^9^, ^10^, ^11^, ^12^, ^13^, ^14^, ^15^, ^16^, ^17^ ^]^ where carbon simultaneously serves as conductive agent, strong adsorbent or even a compartment impeding the dissolution of long‐chain LiPS during cycling. At the cell‐level, the specific energy of S/C composites is limited by 1) carbon “dead weight,”^[^ ^18^, ^19^ ^]^ 2) high carbon porosity, necessitating larger amounts of electrolytes to sufficiently wet the cathode, adding weight to the cell and diminishing the specific energy,^[^ ^19^ ^]^ and 3) low S‐loadings (<5 mg cm^−2^).^[^ ^8^, ^10^ ^]^

**Figure 1:**
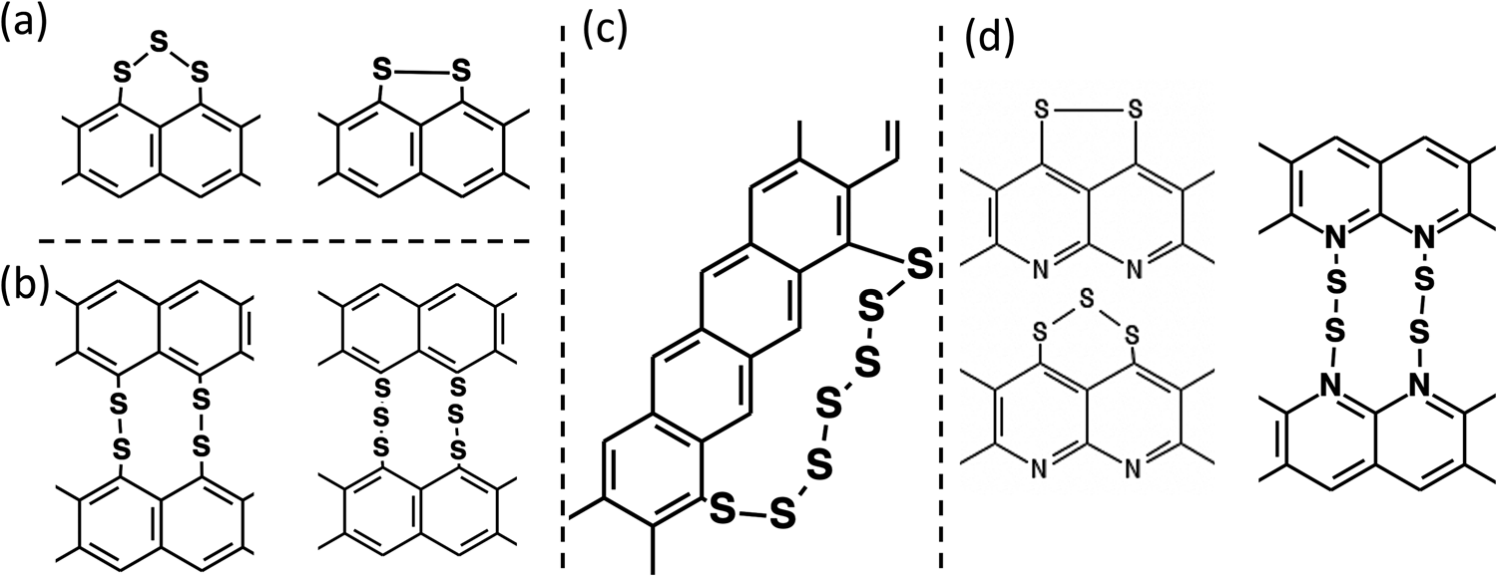
(a) and (b) show possible C—S and S—S bonds with S_2_ or S_3_ chains between different rings in sulfurized polymers with low S content (<35 wt%). c) Longer S chains (S*_x_* with *x* > 3) are inevitably formed at high S content (>35 wt%). d) The presence of N atoms in SP leads to C—N and N—S bonds in specific materials such as sulfurized polyacrylonitrile or SPAN.

Novel sulfurized polymers (SP) in which the sulfur is covalently bound to a carbon network have been shown to deliver high capacities (*at the sulfur level*) with no LiPS formation.^[^ ^4^, ^20^, ^21^, ^22^, ^23^, ^24^, ^25^, ^26^, ^27^, ^28^ ^]^ Among the various SP cathodes, sulfurized poly(acrylonitrile) (labeled as SP‐1N in this article for the ease of discussion and comparison with other SP materials) has attracted much interest due to its excellent electrochemical performance, cycling stability, and low process cost.^[^ ^4^, ^20^, ^21^, ^22^, ^23^, ^24^, ^25^, ^26^, ^27^, ^28^ ^]^ Previously, different molecular structures have been proposed for sulfur incorporation into carbonized polymer backbones using various tools such as electrochemical signatures, nuclear magnetic resonance, X‐ray photoemission, infrared, and Raman spectroscopy.^[^ ^20^, ^21^, ^22^, ^25^, ^29^, ^30^, ^31^, ^32^, ^33^, ^34^, ^35^ ^]^ Based on these findings, it is expected that at low sulfur content (<35 wt%), short sulfur chains (S_2_, S_3_) are linked within one hexagonal carbon ring (**Figure** **1**a) or present as a bridge between two rings (Figure 1b). At higher S content, longer sulfur chains (S*_x_*, *x* > 3) form across the rings (Figure 1c). In SP materials such as SP‐1N, the presence of N atoms in the carbonized polymer backbone results in polypyridine rings (Figure 1d) with N—S bonds. Many different reaction mechanisms have been proposed to explain Li‐SP electrochemistry.^[^ ^20^, ^21^, ^22^, ^25^, ^27^, ^29^, ^30^, ^31^, ^32^, ^33^, ^34^, ^35^ ^]^ These studies suggest that a conjugated structure containing thioradicals is generated from SP via the cleavage of S—S and C—S bonds during the first discharge cycle. The Li^+^ ions then react with anionic sites around S, C, and N atoms (if present) via a reversible lithium‐coupled electron transfer process to form ion‐coordination bonds. A cleavage of the S—S bond results in the formation of Li_2_S and a lithiated backbone via Li—C—N—Li and Li—C—C—Li. Following the first discharge, a substantial fraction of the lithiated Li—C—N—Li and Li—C—C—Li moieties do not undergo delithiation, which leads to an initial irreversible capacity loss after the first cycle.^[^ ^36^, ^37^ ^]^ After this conditioning, a stable cycling is achieved in some SP such as SP‐1N often up to 10 K cycles. Despite this progress, a full understanding of Li‐SP electrochemistry is still missing due to multiple unknown structural features and variations in SP electrochemical behavior resulting from different synthesis conditions.^[^ ^36^, ^37^ ^]^

It has been suggested that at least 70 wt% S with >60% S‐utilization is necessary for realizing practical LSB pouch cells.^[^ ^8^, ^17^ ^]^ Although SP materials like SP‐1N exhibit high capacity (≈13–1400 mAh g_s_^−1^) at the sulfur level, their S content has been limited to 30–35 wt%, which is often said to make them unsuitable for practical LSBs. However, this assumes that the carbon backbone in SP materials does not contribute to the total capacity. Beltran et al.^[^ ^38^ ^]^ theoretically showed that the carbon skeleton in SP materials is not “dead weight” as it exhibits electrochemical activity via multiple C−Li and N−Li interactions mostly with edge/open carbon atoms and pyridinic nitrogen. Building on this, we address the following important questions in Li‐SP electrochemistry that are critical for practical LSBs based on SP materials: 1) Is the carbon skeleton of SP “dead weight” in that it does not significantly contribute to total capacity? 2) In case of an “active” carbon skeleton, does practical realization still require high S content ≈70 wt% in SP cathodes similar to elemental S/C composites? 3) Does N play any role in improving the “activity” of carbon skeleton in N‐containing SP (e.g., SP‐1N)? 4) Can practical S‐loadings >5 mg cm^−2^ (from five‐5s in ref. [8]) of SP materials be achieved with high energy densities (≈350 Wh kg^−1^ at the cell level)?

To address these questions, we synthesized four different SP electrodes (labeled SP‐1N, SP‐1, SP‐2, and SP‐3) with different S (33–70 wt%) and N (≈15 wt%) content. By using in situ Raman spectroscopy, cyclic voltammetry (CV) at different scan rates along, and detailed electrochemical impedance spectroscopy (EIS), we deconvoluted specific surface and redox contributions to the total capacity showing that SP‐1N functions as a pseudocapacitor with an active involvement of the carbon skeleton. SP electrodes containing S content similar to SP‐1N (≈33 wt% S) but without N atoms (labeled SP‐1) showed long cycle stability but three‐times lower capacity suggesting that N atoms are crucial for high capacity. Often, the capacitive contribution of the carbon skeleton is overwhelmed by its low serial quantum capacitance arising from its low density of electronic states at the Fermi level (*E*_F_). The presence of N atoms (e.g., pyridinic) in the carbon skeleton of SP alleviates such low quantum capacitance by increasing the electronic density of states at *E*_F_ (DOS (*E*_F_)) leading to a higher overall capacity.^[^ ^39^ ^]^ Our in situ Raman spectroscopic measurements revealed that C—N and C—C bonds in SP‐1N undergo significant restructuring during the first cycle. SP with higher S content (viz., SP‐2 and SP‐3 with 60 and 70 wt% S, respectively) exhibited poor cycling stability due to the presence of longer S chains. The use of different electrolytes, salt combinations, and additives did not significantly change the electrochemical behavior of SP with high S‐content.

Considering the active nature of carbon skeleton in SP‐1N, we discuss the relevance and appropriateness of capacities calculated on per gram S basis in SP as that may wrongly attribute extra carbon skeleton capacitance to S. The recognition of active carbon skeleton in SP‐1N behooves us to reconsider critical metrics for SP materials separately from conventional S_8_/C composites.^[^ ^8^ ^]^ Based on the energy density goals proposed by the United States Advanced Battery Consortium (USABC) for electric vehicle batteries (350 Wh kg^−1^ at the cell level and 235 Wh kg^−1^ at pack level at C/3 discharge rate), we identified critical metrics for SP‐1N materials with 35–45 wt% S. We show that for SP‐1N with 35 wt% S, S loading >5 mg cm^−2^ with E/S ratio <2 µL mg^−1^ and N/P ratio <5 is necessary for achieving 350 Wh kg^−1^ at the cell level. The required S loading is lower ≈4 mg cm^−2^ if the S content in SP‐1N is increased to 45 wt%. The total cathode mass for 35 wt% S containing SP with ≈5 mg cm^−2^ S loading is more than twice that of 70 wt% S_8_. Although carbon backbone plays an active role, SP electrodes are much thicker and heavier than S_8_ due to such excess weight. This presents a problem in using conventional Al current collectors that cannot support high mass loading due to delamination issues. To overcome this, we developed an “active” carbon nanotube (CNT) bucky sandwich (BS) current collector (amenable for roll‐to‐roll processing) that contributes to the total capacity offsetting its excess weight. By using BS structure instead of conventional Al current collector, we achieved >5 mg cm^−2^ S loading with SP‐1N. At lower sulfur loading of ≈0.4 mg cm^−2^ (≈1.15 mg cm^−2^ of SP‐1N), BS SP‐1N electrode was able to withstand high C‐rates with a gravimetric capacity ≈1300–1400 mAh g_s_^−1^ (≈200 mAh g_electrode_^−1^) after ≈1000 cycles at 2.5C. We were also able to load BS structure with ≈5.5 mg cm^−2^ S (≈15.8 mg cm^−2^ of SPAN), which yielded a sulfur‐level gravimetric capacity ≈1360 mAh g_s_^−1^ (≈690 mAh g_s_^−1^), electrode level capacity 200 mAh g_electrode_^−1^ (100 mAh g_electrode_^−1^), and areal capacity ≈7.8 mAh cm^−2^ (≈4.0 mAh cm^−2^) at 0.1C (1C) rate for ≈100 cycles at E/S ratio = 7 µL mg^−1^. We also succeeded in preparing pouch cells using BS SP‐1N electrode containing ≈5 mg cm^−2^ S with a capacity ≈1300 mAh g_s_^−1^ (≈190 mAh g_electrode_^−1^) at 0.1 C rate.

## 2 Results and Discussion

### 2.1 Physicochemical Characterization

We prepared four different SP materials with different C, S, and N contents, which are labeled as SP‐1, SP‐1N, SP‐2, and SP‐3. The bulk compositional analysis of all samples obtained using CHNS (Carbon, Hydrogen, Nitrogen, and Sulfur) measurement is shown in **Table** **1**.

**Table 1:** Bulk elemental analysis of C, H, N, and S content in all samples

Sample	C [wt%]	N [wt%]	S [wt%]	H [wt%]	Atomic ratio (S/C)	Atomic ratio (N/C)
SP‐1N	40.5	15	34.8	0.9	0.32	0.16
SP‐1	39.3	0	33.3	1.3	0.31	0
SP‐2	26.7	0	61.8	0.2	0.86	0
SP‐3	23.9	0	70.3	0.1	1.1	0

**Figure 2:**
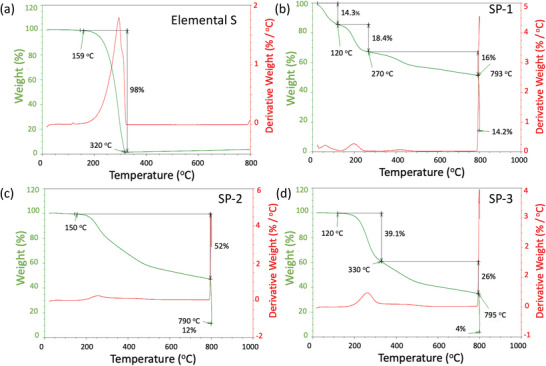
Thermogravimetric profiles for a) elemental S, b) SP‐1, c) SP‐2, and d) SP‐3 samples with different S content. A clear difference is observed between SP‐1 and elemental S with two weight loss steps occurring below 270 °C in SP‐1. This suggests the presence of shorter S chains such as S_2_ or S_3_. While SP‐2 and SP‐3 appear similar to elemental S, the weight loss was observed a prolonged duration up to 790 °C suggesting that there are longer S chains but not in octagonal form (S*_x_*, *x* = 3–7).

Both SP‐1 and SP‐1N possess ≈33–35 wt% of S. While SP‐1N contains ≈15 wt% N (Table 1), SP‐1 has no N content. SP‐2 and SP‐3 have a higher S content of ≈60 and 70 wt%, respectively but do not contain any N. In order to prepare SP‐1 with the same S content as SP‐1N (prepared using polyacrylonitrile or PAN) but without N atoms, we used a different polymer precursor viz., polyphenylene sulfide (PPS). SP‐2 and SP‐3 were also synthesized using PPS. We performed thermogravimetric analysis (TGA) to understand structural differences between SP samples with different S content (**Figure** **2**). SP‐1 showed clearly distinct weight loss features that are different from elemental S (Figure 2a,b).

As shown in Figure 2b, we observed ≈32.7 wt% decrease in weight (*W*) below 270 °C, which is close to the total S content ≈33.3 wt% in SP‐1 (see Table 1). Unlike elemental S, we found ≈14 wt% decrease below 120 °C and ≈18 wt% decrease between 120 and 270 °C for SP‐1. These are seen as two peaks in the derivative curves (d*W*/d*T*) at 50 and 200 °C. Based on the cold crystallization temperature (*T*_c_ = 120 °C) and the melting point (*T*_m_ = 270 °C) of the PPS crystal structure,^[^ ^40^ ^]^ it is likely short S chains in SP‐1 undergo semi‐crystalline transition above *T*_c_ leading to interspersed S atoms forming *φ* − *S* − *φ* bonds (*φ* represents hexagonal carbon, see Figure 1b). While SP‐2 and SP‐3 (Figure 1c,d) profiles appear similar to elemental S, the weight loss was observed for a prolonged duration up to 790 °C suggesting that there are longer S chains but not in octagonal form (S*_x_*, *x* = 3–7). The weight loss for SP‐2 (/SP‐3) between 150 and 790 °C was ≈52 wt% (/≈70 wt%), which is comparable to its S content ≈61.8 wt% (/70.3 wt%) shown in Table 1. Unlike SP‐1, SP‐1N (see Figure S1, Supporting Information) did not show significant weight loss between 120 and 270 °C implying the initial presence of *φ*—*S*—*φ* bonds leading to no further crystallization or S—S cleavage above the glass transition (*T*_g_ = 95 °C) of PAN. This is expected from its low surface area and pore volume (≈18 m^2^ g^−1^ from Brunauer‐Emmett‐Teller or BET surface area measurements), which suggests that SP‐1N is already a closed structure with less porosity.

**Figure 3:**
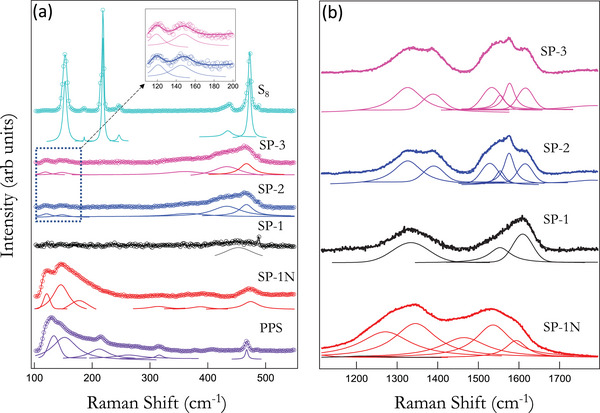
a) Raman spectra of elemental S, polyphenylene sulfide (PPS), and sulfurized polymer (SP) samples in the low frequency region 100–500 cm^−1^. The inset shows a magnified view of the 100–200 cm^−1^ region for SP‐2 and SP‐3 samples. All the spectra were obtained with 532 nm excitation and fit using Lorentzian peaks. The vibrational modes at ≈150 and 220 cm^−1^ represent asymmetric and symmetric S—S bending in S_8_ respectively while the peaks at 435 and 470 cm^−1^ are related to S—S stretching modes in long chain S and S_8_.^[^ ^33^, ^35^ ^]^ SP‐1 (≈35 wt% S without N atoms) shows a weak and broad feature ≈475 cm^−1^ suggesting the absence of long chain S, which was also evident from thermogravimetric analysis in Figure 2. SP‐2 (60 wt% S) and SP‐3 (70 wt% S) show strong 435 and 470 cm^−1^ features with the peak area ratio *A*_435_/*A*_475_ >2 indicating long chain S. In PPS, the sharp peak observed ≈475 cm^−1^ was previously assigned to *φ* − *S* deformation and out‐of‐plane vibrations.^[^ ^3^ ^]^ A similar peak was found in SP‐1N. By combining TGA and Raman spectroscopic data, it is seen that no S_8_ is present in SP samples although long chain S*_x_* (*x* < 8) are predominant in SP‐2 and SP‐3. b) Raman spectra for all SP samples showed typical carbon skeleton signatures in 1200–1650 cm^−1^ region. Specific peak positions and full widths were obtained by fitting the spectrum (see Table 2). SP‐2 and SP‐3 showed a rich carbon peak structure similar to SP‐1N suggesting they contain sp^2^/sp^3^ carbon clusters. Two major differences between SP‐1N and SP‐1‐3 samples are: 1) a downshifted G band ≈1575 cm^−1^ suggesting more sp^2^ content in SP‐1‐3 and 2) the presence of D’ band ≈1615 cm^−1^ indicating open pores with armchair like defects in SP‐1‐3.

We obtained the Raman spectra of S_8_ and PPS to study different signatures for short/long S chains and *φ* − *S_x_* vibrations (*cf*. Figure 1). As shown in **Figure** **3**a, the Raman spectrum for elemental S_8_ exhibited three sharp and prominent peaks at 150, 220, and 470 cm^−1^. A relatively weaker and broader peak is also observed at ≈435 cm^−1^. The vibrational modes at ≈150 and 220 cm^−1^ represent asymmetric and symmetric S—S bending in S_8_, respectively.^[^ ^33^, ^35^ ^]^ The peak at 470 cm^−1^ is related to S—S stretching modes in S_8_.^[^ ^33^, ^35^ ^]^ Previously, Nims et al.^[^ ^41^ ^]^ and Kozhevnikov et al.^[^ ^42^ ^]^ used temperature dependent Raman spectroscopy to show that features at 435 and 470–75 cm^−1^ are associated with long S chains (different from S_8_) in liquid sulfur. They found that increasing temperature results in increased intensity of 435 cm^−1^ feature along with the broadening of 470–75 cm^−1^ due to long chain polymerization of S. In addition to S_8_ spectrum, we also collected the Raman spectrum of PPS to identify *φ* − *S* vibrations.^[^ ^43^ ^]^ The sharp peak observed ≈475 cm^−1^ was previously assigned to *φ* − *S* deformation and out‐of‐plane vibrations in PPS.^[^ ^43^ ^]^ Two lower frequency features ≈129 and 145 cm^−1^ are similar to the so‐called “butterfly mode” where two adjacent rings bend in unison mimicking a butterfly in flight.^[^ ^43^ ^]^ Based on these mode assignments, we analyze the Raman spectra of SP samples below.

We found that SP‐1N showed a spectrum similar to PPS in 100–500 cm^−1^ region suggesting that SP‐1N contains *φ* − *S* and *φ* − *S_x_* (*x* ≥ 2) bonds similar to PPS. The complete formation of these bonds is also reflected in low surface area (≈18 m^2^ g^−1^) of SP‐1N compared other S/C composites (>100 m^2^ g^−1^). In addition to the 475 cm^−1^ peak, two broad and weak features ≈305 and 375 cm^−1^ are also observed in SP‐1N. These were previously identified with out‐of‐plane vibrations of N atoms in C—N and the twisting of the carbon backbone.^[^ ^35^ ^]^ Unlike SP‐1N, all these features are absent in SP‐1 indicating the absence of *φ* − *S_x_* (*x* > 2) bonds. This concurs with the thermogravimetric profile of SP‐1 exhibiting weight loss above *T*_c_ (>120 °C) upon the formation *φ* − *S_x_* (*x* > 2) bonds. A broad Raman feature ≈450 cm^−1^ in SP‐1 suggests the presence of C—S bonds.^[^ ^35^ ^]^ SP‐2 and SP‐3 samples showed three overlapping prominent broad peaks ≈365, 435, and 475 cm^−1^. The peak ≈365 cm^−1^ arises from carbon chain deformation in the backbone^[^ ^35^ ^]^ while 435 and 475 cm^−1^ peaks have been attributed to long S chains.^[^ ^41^ ^]^ The downshift in carbon deformation peak in SP‐2 and 3 maybe attributed to higher sp^2^ content compared to SP‐1N. The integrated intensity (denoted by *A* or peak area) ratio *A*_435_/*A*_475_ for SP‐2 and SP‐3 was >2 implying that SP samples contain more S in the form of longer S chains at higher S content (≈61 and 70 wt% S in SP‐2 and SP‐3, respectively). SP‐1N only exhibited a weak 475 cm^−1^ peak without a lower shoulder ≈435 cm^−1^ suggesting that 475 cm^−1^ in SP‐1N is related more closely to *φ* − *S* vibrations similar to PPS rather than S—S stretching. A broad peak ≈305 cm^−1^ (attributed to C—N vibrations in SP‐1N^[^ ^35^ ^]^) was absent in SP‐2 and SP‐3. Although much weaker than 435–475 cm^−1^ features, SP‐2 and SP‐3 also exhibited lower frequency features ≈120 and 140 cm^−1^ similar to SP‐1N and PPS. In summary, our SP samples contain different chain lengths of S with more longer S chains at higher S content (SP‐2 and SP‐3). Both TGA and Raman confirm that no SP samples contained S in elemental S_8_ form.

Raman spectroscopy also provides many useful insights into the structure of the carbon skeleton in SP materials. Generally, the Raman spectrum of carbon compounds exhibits several features in 1000–1700 cm^−1^ region such as the disorder (*D*), graphitic (*G*), and the nongraphitic defect (*D*′) bands.^[^ ^44^ ^]^ The peak positions for these bands depend upon multiple factors such as carbon hybridization (sp^2^ vs sp^3^), crystallinity, layer stacking (e.g., graphite/graphene vs carbon nanotubes), defect configuration, and the excitation wavelength. Raman spectra for all SP samples showed typical carbon skeleton signatures in 1200–1650 cm^−1^ region (Figure 3b). Specific peak positions and full widths were obtained by fitting the spectrum (see **Table** **2**).

**Table 2:** Raman spectral features and their assignments in all SP samples

Sample	Peaks [cm^−1^]	Assignment
SP‐1N	305	C—N out‐of‐plane vibrations^[^ ^35^ ^]^
	375	Carbon backbone twisting^[^ ^35^ ^]^
	475	*φ* − *S_x_* deformation where *φ* is carbon ring and S—S stretch^[^ ^43^ ^]^
	1280	D‐band in stage 3 amorphous carbon with >20% sp^3[^ ^44^, ^66^, ^67^, ^68^ ^]^
	1350	Disorder or D‐band in microcrystalline graphite^[^ ^44^, ^66^, ^67^, ^68^ ^]^
	1464	A‐band in stage 3 amorphous carbon with >20% sp^3[^ ^44^, ^66^, ^67^, ^68^, ^69^ ^]^
	1534	G‐band in stage 3 amorphous carbon with >20% sp^3[^ ^44^, ^66^, ^67^, ^68^ ^]^
	1592	G‐band in microcrystalline graphite
SP‐1	450	C—S*_x_* bonds^[^ ^41^, ^42^ ^]^
	1335	D‐band similar to activated carbon^[^ ^44^, ^66^, ^67^, ^68^ ^]^
	1564	G‐band similar to activated carbon^[^ ^44^, ^66^, ^67^, ^68^ ^]^
	1611	D’‐band similar to activated carbon^[^ ^44^, ^66^, ^67^, ^68^ ^]^
SP‐2 and SP‐3	365	Carbon backbone twisting^[^ ^35^ ^]^
	435	S—S stretching in long chain S^[^ ^35^ ^]^
	475	*φ* − *S_x_* deformation^[^ ^43^ ^]^
	1340	D‐band in stage 2 amorphous carbon with 5–10% sp3^[^ ^44^, ^66^, ^67^, ^68^ ^]^
	1398	G‐band in stage 2 amorphous carbon with 0–5% sp3^[^ ^44^, ^66^, ^67^, ^68^ ^]^
	1545	G‐band in stage 2 amorphous carbon with 5–10% sp3^[^ ^44^, ^66^, ^67^, ^68^ ^]^
	1575	G‐band in stage 2 amorphous carbon with 0–5% sp3^[^ ^44^, ^66^, ^67^, ^68^ ^]^
	1615	D’‐band in nanocrystalline graphite^[^ ^44^, ^66^, ^67^, ^68^ ^]^

SP‐1N spectrum showed five broad peaks ≈1280, 1350, 1464, 1535, and 1592 cm^−1^. While the bands ≈1280 and 1350 cm^−1^ correspond to the D‐band in sp^2^ carbon, the bands ≈1464 and ≈1535 cm^−1^ are assigned to sp^2^/sp^3^ carbon clusters. Previously, we found that nongraphitic dopants and open pores are akin to armchair defects in that they lead to a strong *D*’‐band in graphitic sp^2^ carbon.^[^ ^45^ ^]^ Although a strong D‐band was present in SP‐1N, we did not observe a *D*’‐band (≈1610–1620 cm−^1^) despite the presence of nongraphitic N dopants. This is attributed to the closed structure of SP‐1N with low porosity (≈0.04 cc g^−1^ for pores <20 nm). SP‐1N exhibited an upshifted G‐band ≈1592 cm^−1^ compared to SP‐2 and SP‐3 (≈1575 cm^−1^). Such upshift is commonly observed due to curvature in closed carbon structures such as fullerenes and carbon nanotubes. The absence of *D*’ band and upshifted *G* band suggest a closed structure without many open pores within SP‐1N. This concurs with the low surface area ≈18 m^2^ g^−1^ observed for SP‐1N. SP‐1 showed features similar to activated carbon (AC) with D‐band ≈1335 cm^−1^, G‐band ≈1564 cm^−1^, and *D*’‐band ≈1611 cm^−1^ plausibly due to open carbon rings with no long range order. A comparison of SP‐1 and AC spectra is presented in Figure S2 in the Supporting Information. SP‐2 and SP‐3 showed a rich carbon peak structure similar to SP‐1N suggesting they contain sp^2^/sp^3^ carbon clusters (see Table 2 for detailed assignments). Two major differences between SP‐1N and SP‐1‐3 samples are: 1) a downshifted G band ≈1575 cm^−1^ suggesting more *sp* ^2^ content in SP‐1‐3 and 2) the presence of *D*’ band ≈1615 cm^−1^ indicating open pores with armchair like defects in SP‐1‐3.

We observed that the x‐ray photoemission spectroscopy (XPS) for all samples showed a clear presence of C—S—C bonds corresponding to ≈164 eV (see Figure S3a, Supporting Information). In case of SP‐1N, we found some H—S—C bonds at ≈161.6 eV. SP‐1N, SP‐2, and SP‐3 also displayed a broad peak ≈168.5 eV, which is related to sulfone (C‐SO_2_). A detailed discussion on the influence of sulfone groups on electrochemistry is presented later in Figure S11 in the Supporting Information. Interestingly, this peak was more intense SP‐1 and was upshifted ≈170 eV suggesting the presence of SO_4_^2−^ on the surface. The configuration of N atoms in SP‐1N can be gleaned from Figure S3b in the Supporting Information. The peak ≈ 400 eV is indicative of majorly pyridinic N in the carbon skeleton of SP‐1N.

### 2.2 Electrochemical Performance

**Figure 4:**
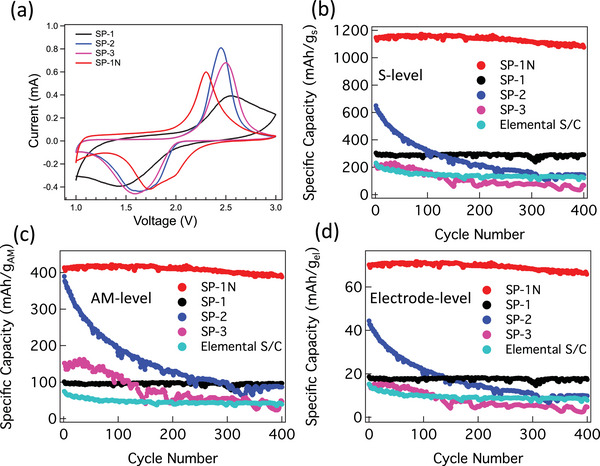
a) Cyclic voltammetry of all SP electrodes performed at a low scan rate ≈0.1 mV s^−1^. The redox peaks observed for different samples are listed in Table 3 along with the values for S_8_/C electrodes. Unlike elemental S, CV scans for SP samples showed one or two cathodic peaks at lower voltages between 1.5 and 2.0 V. The first cathodic peak in SP‐1 was broad and less intense compared to SP‐2, 3, and 1N. The complete absence of any cathodic peaks ≈2.35 V in all SP samples suggests that the reduction mechanism in all SP samples neither starts from S_8_ nor via the formation of S*_x_* ^2−^ with *x* ≥ 4.^[^ ^4^, ^5^ ^]^ All SP samples exhibited the second cathodic peak at 1.5–1.7 V (Table 3), which is related to the formation of Li_2_S. SP‐1, 2, and 3 showed similar cathodic peak structure but were downshifted compared to SP‐1N suggesting differences in their mechanisms. During oxidation, a maximum at ≈2.3–2.5 V occurs for S oxidation. The oxidation peak is upshifted for SP‐1, 2, and 3 possibly due to different S chain length. (b)–(d) show specific capacity of coin cells made using SP materials coated on Al/C current collector at S loading ≈0.4 mg cm^−2^ with Li foil anode. Different normalizations using S, active material (AM), or the total electrode mass level are shown for appropriate comparison. While SP‐1 and SP‐1N showed good stability, the specific capacity of SP‐2 and 3 rapidly deteriorated similar to elemental S/C. Given that SP‐1 contains same S content as SP‐1N without any N atoms, it is likely that N atoms are not need for achieving cycling stability in SP materials. However, SP‐1N showed a significantly higher specific capacity at all levels indicating the direct involvement of both carbon backbone and N atoms in electrochemical reactions.

To evaluate the electrochemical performance, CV was performed on all samples (**Figure** **4**a). The redox peaks observed for different samples are listed in **Table** **3** along with the values for elemental S/C electrodes.

**Table 3:** Redox peaks for elemental S and SP cathodes

Sample	Anodic peak [V]	Cathodic Peak 1 [V]	Cathodic Peak 2 [V]
SP‐1N	2.3	2.0	1.7
SP‐1	2.5	NA	1.5
SP‐2	2.45	1.8	1.5
SP‐3	2.5	1.8	1.6
Elemental S/C	2.42	2.35 (Li_2_S_8,_ Li_2_S_4_)	2.05 (Li_2_S_2_)

In case of elemental S/C electrodes,^[^ ^30^, ^37^ ^]^ a moderately intense cathodic peak ≈2.35 V corresponding to long chain polysulfides, i.e., Li_2_S_8_ and Li_2_S_4_ is observed along with another maximum at 2.05 V related to the formation of Li_2_S_2_ (Table 3). During oxidation, a maximum at ≈2.42 V occurs for S oxidation. Unlike elemental S, CV scans for SP samples showed either one or two cathodic peaks at lower voltages between 1.5 and 2.0 V. The complete absence of any cathodic peaks ≈2.35 V in all SP samples suggests that the reduction mechanism neither starts from S_8_ nor via the formation of S*_x_* ^2−^ with *x* ≥ 4.^[^ ^30^, ^37^ ^]^ This further supports our conclusions from TGA and Raman studies that no octagonal sulfur is present in SP samples. Fanous et al.^[^ ^30^, ^37^ ^]^ previously hypothesized that polymer‐bound sulfur chains in SP cathodes are first reductively broken to form terminal SP−S*_x_*‐S^−^ moieties. These are then degraded stepwise from the chain end with concomitant formation of Li_2_S until the sulfur chain is completely reduced according to SP−S*_x_* ^−^ + 2Li^+^ + 2e^−^ → SP−S*_x_*_−1_^−^ + Li_2_S (starting from 2 ≤ *x* ≤ 7 and ending at *x* = 2). All SP samples exhibited a cathodic peak at 1.5–1.7 V (Table 3), which is related to the formation of Li_2_S. SP‐1, 2, and 3 showed similar cathodic peak structure but were downshifted compared to SP‐1N. The first cathodic peak in SP‐1 was broad and less intense compared to SP‐2, 3, and 1N.

S atoms in a double bond (e.g., >C=S and —C(=S)—S—) are electrochemically inactive while the activity of C—S single bond depends upon the stability of either the radical or the carbanion formed by the elimination of S^[^ ^20^, ^46^ ^]^ (Equations (1) and (2))

(1) $$-C-\mathrm{SLi}+{\mathrm{Li}}^{+}+e^{-}\to -C\cdot +{\mathrm{Li}}_{2}S$$

(2) $$-C-\mathrm{SLi}+2{\mathrm{Li}}^{+}+2e^{-}\to -C-{\mathrm{Li}}^{+}+{\mathrm{Li}}_{2}S$$

In case of sp^3^ carbon, C—S bond is inactive as the radical/carbanion formed in Equations (1) and (2) cannot be stabilized. But, C—S bond becomes electrochemically active when S is bound to a conjugated sp^2^ carbon, which is able to delocalize the radical and carbanion.^[^ ^46^ ^]^ The reduction potentials of Equations (1) and (2) decrease with the total degree of delocalization (≈1.3 V vs Li/Li+ for long linear polyacetylene, 0.1–0.3 V vs Li/Li+ for large plane graphite).

The cathodic peaks in SP‐1‐3 could be downshifted due to a higher degree of delocalization compared to SP‐1N. This is inferred from Raman spectra presented in Figure 3 and Table 2. Based on three stages of amorphous carbon proposed by Ferrari et al. using Raman spectroscopy,^[^ ^44^ ^]^ we identified that the SP‐1N carbon backbone has more stage 3 diamond‐like amorphous carbon while SP‐1,2, and 3 contained stage 2 micro and nanocrystalline graphitic carbon (Table 2). The oxidation peak at 2.3 V corresponds to the formation of sulfur chains in SP according to SP−S− Li^+^ + *x* Li_2_S → SP−S*_x_*_+1_ − Li^+^ + 2*x* Li (1 ≤ *x* ≤ 7). In case of SP‐1‐3, we observed upshifted anodic peaks compared to SP‐1N, which is also attributed to the differences in the carbon backbone.

### 2.3 In Situ Raman Spectroscopy

**Figure 5:**
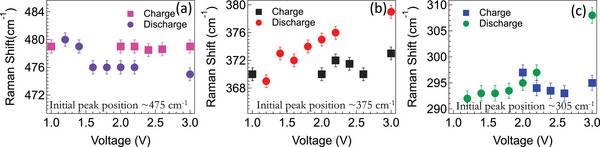
In situ Raman spectroscopy was performed on SP‐1N during the first cycle to understand the role of N atoms and the carbon backbone. The variation in peak positions during first charge/discharge are shown for a) ≈475 cm^−1^ (related to S—S bonds), b) ≈375 cm^−1^ (carbon backbone), and c) ≈305 cm^−1^ (C—N bonds).

**Figure 6:**
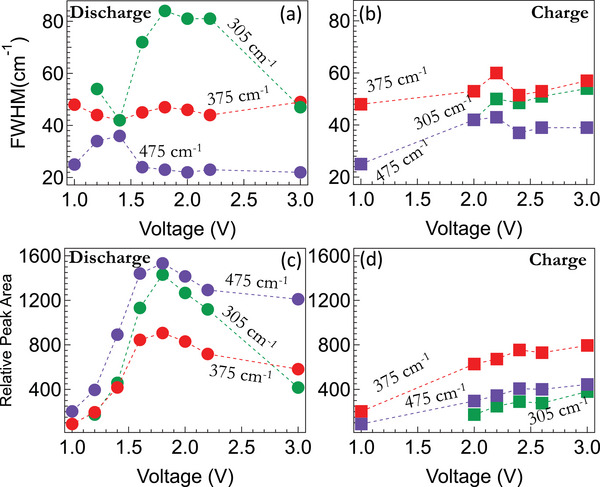
Three peaks ≈475 cm^−1^ (related to S—S bonds), ≈375 cm^−1^ (carbon backbone), and ≈305 cm^−1^ (C—N bonds) were studied using in situ Raman spectroscopy during first charge/discharge. The variation in full width at half‐maximum (FWHM) for all peaks during first a) discharge and b) charge. The relative peak areas for all peaks during first c) discharge and d) charge. The observed changes suggest that the sp3 clusters in SP‐1N open up during the first cycle leading to an active involvement of the carbon backbone in subsequent cycles.

The cycling data for all SP samples is shown in Figure 4b–d with different mass normalizations (viz., S, SP, and total electrode mass). We observed that SP‐1N and SP‐1 (containing ≈33–35 wt% S) showed stable cycling unlike SP‐2, SP‐3, and elemental S/C cathode. The rapid degradation in SP‐2 and SP‐3 with higher S loadings is attributed to longer ring‐bound S chains that result in the loss of active material during oxidation similar to elemental sulfur (cf. Figure 1c). Although SP‐1N and SP‐1 contain similar amounts of S and exhibit similar stability, SP‐1N showed a significantly higher capacity than SP‐1 implying that carbon backbone and N atoms play an important role in the total capacity. We hypothesize that C—N and C—C bonds are opened up in first cycle of SP‐1N for stabilizing the radical and carbanion (in Equations (1) and (2)). To validate this, we investigated the role N and carbon backbone using in situ Raman spectroscopy of SP‐1N at 1st and 52nd cycle. We observed important changes in low frequency peaks at 305, 375, and 475 cm^−1^ (**Figures** **5** and **6** and Figure S4, Supporting Information). It should be recalled that 305 and 375 cm^−1^ peaks are related to C—N out‐of‐plane vibrations and carbon backbone twisting, respectively.^[^ ^35^ ^]^ The peak ≈475 cm^−1^ in SP‐1N is related to *φ* − *S_x_* deformation and S—S stretching. As shown in Figures 5 and 6, all three peaks were found to change in their position, area, and full width at half maximum (FWHM). Specifically, the peak position (Figure 5) and FWHM of 305 and 375 cm^−1^ peaks (Figure 6) showed a clear association with the first cathodic peak ≈2 V during first discharge. While the peak position of 305 and 375 cm^−1^ downshifted by ≈5–10 cm^−1^ between 3.0 and 2.0 V during the first discharge, the third peak ≈475 cm^−1^ did not change significantly <1 cm^−1^ (Figure 5a–c). The FWHM shows a clear peak ≈2 V for 305 and 375 cm^−1^ while a peak ≈1.5 V is observed in 475 cm^−1^.

**Figure 7:**
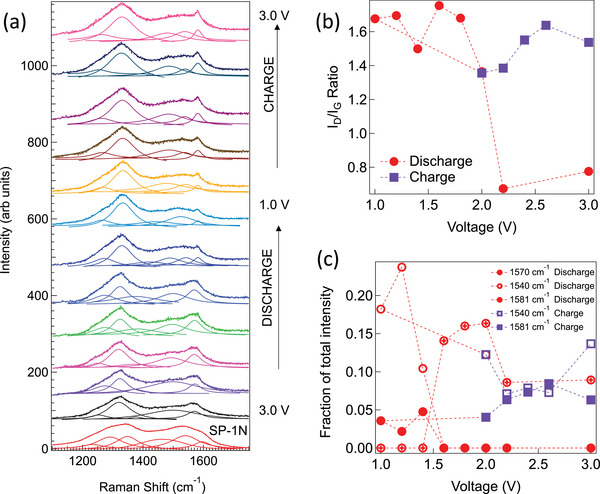
a) In situ Raman spectra showing changes in the carbon backbone (1100–1800 cm^−1^ region) during the first charge/discharge cycle. b) The ratio of defect‐induced band (D‐band) to graphitic band (G‐band), known as the *I*_D_/*I*_G_ ratio, indicates the density of defects with higher defect density at higher *I*_D_/*I*_G_. We observed significant changes in *I*_D_/*I*_G_ ratio below 2 V during first discharge (concomitant with cathodic peaks in Figure 3a) and ≈2.5 V during first charge (concomitant with anodic peak in Figure 3a). c) The peaks in 1500–1600 cm^−1^ region are strongly dependent upon the hybridization and topology of carbon network. Similar to the *I*_D_/*I*_G_ ratio, we found that the fraction of total intensity for different peaks changed differently during the first cycle indicating the active participation of carbon backbone.

During the first discharge cycle, FWHM was observed to change mainly for the 305 cm^−1^ peak (Figure 6a). The variation in relative peak area and FWHM during first charge was much less than the first discharge, which was also the case for 52nd cycle (Figure S5, Supporting Information). Given the significant changes in 305 and 375 cm^−1^ that are related to the carbon skeleton, we also investigated the spectral changes in 1200–1600 cm^−1^ region to identify changes in sp^2^/sp^2^ carbon. The sp^3^/curved carbon features found in pristine SP‐1N ≈1535 and 1592 cm^−1^ were not found in the electrode at the initial voltage of 3.0 V (**Figure** **7**a). Interestingly, there was only one Raman peak ≈1570 cm^−1^ (instead of two peaks) during the first discharge up to ≈1.5 V.

This suggests that sp^3^ bonds are plausibly broken during the first discharge and allow for Li–S interaction. The ratio of D‐ to G‐band intensity (*I*_D_/*I*_G_), which is directly proportionate to the defect density, changed significantly with a sharp increase near the first cathodic peak in the first discharge cycle (Figure 7b). Upon charging, *I*_D_/*I*_G_ ratio showed a peak ≈2.5 V corresponding to S oxidation. A new peak ≈1581 cm^−1^ corresponding to sp^2^ carbon emerged concomitant with the second cathodic peak ≈1.5 V and remained intense even after the first charge to 3.0 V (Figure 7c). Below 1.5 V, the original peak ≈1540 cm^−1^ in SP‐1N was recovered and remained visible upon charging to 3.0 V although at a lower intensity. The changes in carbon skeleton structure from both low‐ and high‐frequency Raman regions imply that the carbon structure of SP‐1N undergoes significant changes in the first cycle, which plays an important role in its electrochemical stabilization of the carbanion/radical in Equations (1) and (2). As discussed below, the active carbon backbone in SP‐1N boosts the capacitive contributions (arising from −C−Li^+^ and −C—N−Li^+^) in addition to redox active component from S (see Equation (2)). A more detailed Raman analysis of different cycles and C‐rates will be published separately.

### 2.4 Bulk and Surface Contributions

**Figure 8:**
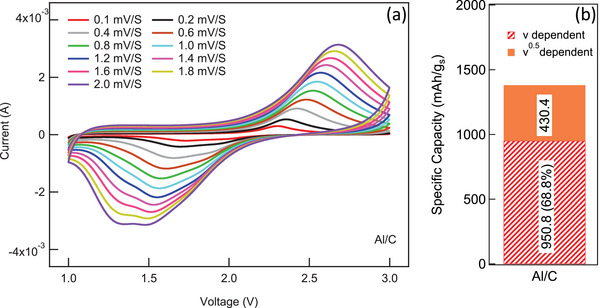
a) Scan‐rate dependent cyclic voltammetry of SP‐1N on Al/C (0.4 mg cm^−2^), b) By using Trasatti's method, capacitive and redox contributions were identified for SP‐1N on Al/C. The involvement of carbon backbone along with the presence of N atoms leads to high capacitive contribution ≈950 mAh g_s_^−1^.

Based on Equations (1) and (2) and the above in situ Raman analysis, we hypothesized that the carbon skeleton in SP‐1N contributes significantly to the total capacity through surface‐dominant electric double layer. To validate this, we quantified the contribution of electric double layer using CV at different scan rates (**Figure** **8**a). The formation of electrical double layer is a surface‐dominant mechanism that is faster than the bulk redox processes. We used Trasatti's method^[^ ^47^, ^48^ ^]^ for the deconvolution of the total, surface, and bulk charges. Assuming that sufficient time is allowed for every reaction to take place, the total charge is the charge related to the full capacity obtained at an infinitesimally small scan rates ≈0 mV s^−1^. As discussed in refs. [47, 48], this can be expressed as

(3) $$\frac{1}{Q\left(v\right)}=\frac{1}{Q_{v=0}}\;+\alpha \sqrt{v}$$

In Equation (3), *Q*(*v*) is the observed capacity at a scan rate *v*, *Q*_*v* = 0_ is the capacity when the scan rate is infinitesimally small ≈0 mV s^−1^, and *α* is a constant. It can be seen that $\frac{1}{Q(v)}$ versus $\sqrt{v}$ is a straight line with *α* as the slope and $\frac{1}{Q_{v\;=\;0}}$ as the *y*‐intercept. Thus, the inverse of the *y*‐intercept of $\frac{1}{Q(v)}$ versus $\sqrt{v}$ plot yields the total charge *Q*_*v* = 0_. On the other hand, at very high scan rates (potentially tending to infinity), the only contribution is from surface charges. This is expressed as a different phenomenological equation shown below.

(4) $$Q\;\left(v\right)=Q_{v\to \infty }\;+\frac{\beta }{\sqrt{v}}$$

In Equation (4), *Q*_*v* → ∞_ is the capacity at very high scan rates and *β* is a constant. As noted in ref. [40], Equations (3) and (4) are analytically not directly related because clearly $\frac{1}{Q_{v\;=\;0}}\neq \frac{1}{Q_{v\to \infty }}$. The *y‐*intercept of *Q*(*v*) versus $\frac{1}{\sqrt{v}}$ yields the surface charge *Q*_*v* → ∞_. The bulk charge is calculated by subtracting the surface charge from total charge. As shown in Figure 8b, we found that SP‐1N on conventional Al/C current collector exhibited ≈69% capacitive and ≈31% redox contributions (plots for Equations (3) and (4) are presented later in Figure 12). The % redox capacity ≈420 mAh g_s_^−1^ concurs with ≈35 wt% S in SP‐1N with 72% utilization (0.72*0.35*1672 mAh g_s_^−1^ = 420 mAh g_s_^−1^). Unlike elemental S/C, the carbon skeleton in SP‐1N plays an active role by adding ≈950 mAh g_s_^−1^ capacity through electric double layer according to Equation (2). Such pseudocapacitive behavior was also observed in the electrochemical impedance spectra or EIS (discussed later in Figure 13). It should be noted that the capacities here are normalized by S mass (in line with the literature on SP) although the pseudocapacitive behavior involves carbon mass. Accordingly, capacity normalized by active material (total SP mass including carbon backbone) and total electrode (SP‐1N, binder, additive, and current collector) mass are presented in Figure 4b–d for all SP samples for completeness. The active role of carbon skeleton implies challenges in comparing different capacity normalizations (viz., per gram sulfur, total material, or electrode), which are discussed later in the next section.

Although SP‐1 showed stability similar to SP‐1N, its total capacity ≈300 mAh g_s_^−1^, which is equivalent to 35 wt% elemental S with 50% utilization. We attribute this reduced capacity to the absence of N in SP‐1. Given that SP‐1 and SP‐1N exhibit only minute decrease in total capacity up to 400 cycles (Figure 4b), it is unlikely that N atoms are necessary for either alleviating polysulfide formation or enabling long cyclability. The observed stability in SP‐1 and SP‐1N is mainly due to the presence of only short S chains in SP‐1 and SP‐1N (Figure 1a,b,d). When S chain length in SP increases at higher S content (SP‐2 and SP‐3), a rapid capacity degradation occurs due to the loss of active material from the formation of higher order polysulfides (Figure 4b). Here, we posit that N atoms in SP‐1N increase the overall quantum capacitance (*C*_Q_) of the carbon backbone in SP‐1N. The capacitive contributions in SP‐1 are dwarfed by a small serial *C*_Q_ imposed by its amorphous carbon backbone. *C*_Q_ arises in series with the double layer capacitance (*C*_dl_) when the electrodes have low electronic DOS(*E*_F_).^[^ ^39^ ^]^ In a conventional parallel plate capacitor with metal electrodes, the adsorption of charge on the metal surface does not shift the chemical potential (*μ*) due to large DOS(*E*_F_). Unlike metals, SP electrodes behave like a semi‐conductor. Moving N electrons (*q* = *Ne*) to a semi‐conductor electrode changes the potential $\Delta V\;=\frac{q}{C_{\mathrm{dl}}}\;$, which is accompanied by an additional change in the chemical potential of the semi‐conductor $\Delta \mu \;=\frac{N}{\mathrm{DOS}(E_{F})}\;=\frac{q}{e.\mathrm{DOS}(E_{F})}\;$. When DOS(*E*_F_) is high, Δ*μ* is close to zero. In case of low DOS(*E*_F_), as in SP electrodes, a nonzero Δ*μ* appears as an extra voltage drop as shown below.

(5) $$\begin{array}{ccc} \Delta \;V_{\mathrm{total}} & = & \frac{q}{C_{\mathrm{device}}}\;=\frac{q}{C_{\mathrm{dl}}}\;+\;\frac{\Delta \mu }{e}=\frac{q}{C_{\mathrm{dl}}}\;+\;\frac{q}{e^{2}.\mathrm{DOS}\left(E_{F}\right)}\\ & = & \;q\left(\frac{1}{C_{\mathrm{dl}}}+\frac{1}{C_{Q}}\right) \end{array}$$

In Equation (5), *C*_Q_ = *e* ^2^ .DOS(*E*_F_) is the quantum capacitance that appears in series and *C*_device_ is the total observed device capacitance. At low DOS(*E*_F_), the total capacitive contribution is dwarfed by very low *C*_Q_. Previously, we demonstrated that the addition of N dopants to nanocarbon (single/few‐layer graphene and carbon nanotubes) leads to an increase in DOS(*E*_F_) and thus alleviates the limitations imposed by low *C*_Q_.^[^ ^39^ ^]^ Although SP‐1N and SP‐1 contain same amount of S, the capacitive contribution in SP‐1 was minimal limiting it to only redox contributions ≈300 mAh g_s_^−1^. In case of SP‐1N, it is likely that N atoms increase DOS(*E*_F_) and *C*_Q_ leading to a significant capacitive contribution from carbon backbone (≈950 mAh g_s_^−1^ as shown in Figure 8b).

### 2.5 Critical Metrics and Capacity Normalization Issues in SP Electrodes

Previously, Manthiram et al.^[^ ^8^ ^]^ established critical metrics known as the “five 5s” for elemental S/C cathodes. Considering that SP cathodes (e.g., SP‐1N) can exhibit both redox and capacitive contributions (cf. Figure 8b), the carbon backbone does not act as dead weight unlike S/C cathodes. Therefore, it is necessary to reevaluate the critical metrics from the standpoint of SP‐1N cathodes by including the capacitive contribution. Although normalization by sulfur mass (*g*_s_) may have some use in evaluating the performance of SP electrodes, the capacities must be normalized by both AM mass (*g*_AM_) and the total cathode mass (*g*_electrode_) for proper comparison with S/C cathodes (Figure 4b–d). We calculated the cell level specific energy for SP‐1N (containing 35 wt% S along with capacitive contributions) and S/C cathodes (70 wt% S with 60% utilization according to ref. [8]) at different S mass loadings and electrolyte‐to‐sulfur (E/S) ratios using the following equation

(6) $${\mathrm{SE}}_{\mathrm{Cell}}=\frac{C_{\mathrm{Total}}V}{M_{\mathrm{Al}}+M_{\mathrm{cm}}+M_{\mathrm{el}}+M_{\mathrm{Li}}+M_{\mathrm{Cu}}+M_{\mathrm{se}}}\;$$

In Equation (6), *C*_Total_ is the total capacity in mAh, *V* is the cell voltage (2.1 V), *M*_Al_ is the aluminum current collector mass, *M*_cm_ is the total cathode material mass, *M*_el_ is the total electrolyte mass, *M*_Li_ is lithium mass (calculated from the corresponding sulfur loading and cathode capacity as discussed in ref. [8]), *M*_Cu_ is the copper foil mass, and *M*_se_ is the separator mass. A detailed list of parameters is provided in Table S1 in the Supporting Information. We also calculated the specific capacity at different levels by normalizing the total capacity with sulfur, AM, and the total electrode mass as follows

(7) $$C_{S}=\frac{C_{\mathrm{Total}}}{M_{s}}\;C_{\mathrm{am}}=\frac{C_{\mathrm{Total}}}{M_{\mathrm{am}}}\;C_{\mathrm{el}}=\frac{C_{\mathrm{Total}}}{M_{\mathrm{Al}}+M_{\mathrm{cm}}}\;$$

For S_8_ cathodes, *C*_Total_ was calculated using *C*_Total_ = *M*_S_ *UC*_Theoretical_, where *M*_S_ is the total sulfur mass in 1 cm^2^ area, *U* is the utilization fraction for S_8_ (fixed at 60%), *C*_Theoretical_ is *S*_8_ theoretical capacity ≈1672 mAh *g*_s_^−1^, For SP cathodes, *C*_Total_ was calculated as *C*_Total_ = *M*_S_ (*C*_R_ + *C*_C_) to include both redox (*C*_R_ ≈ 400 mAh g_s_^−1^) and capacitive (*C*_C_ ≈ 900 mAh g_s_^−1^) contributions that were experimentally identified (cf. Figure 8b). It should be noted that the normalization factor is ultimately canceled out in calculating *C*_Total_ resulting in the total capacity in mAh. *M*_Li_ was calculated using theoretical capacity ≈1672 mAh g_s_^−1^ for S_8_, ≈1300 mAh g_s_^−1^ for SP‐1N, and 3860 mAh g_Li_^−1^ for Li. Different S loadings (116 mg cm^−2^), E/S ratios (1–10 µL mg^−1^), and negative‐to‐positive (N/P) capacity ratio (1–10) were used for all calculations.

**Figure 9:**
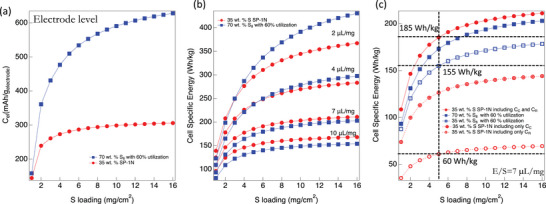
a) Theoretical specific capacity at the electrode level comparing 70 wt% S_8_ (60% utilization) with 35 wt% SP‐1N as a function of total S loading. Capacity is normalized by the total electrode mass. b) Cell specific energy for 70 wt% S_8_ and SP‐1N at different E/S ratios as a function of S loading. Energy is normalized by the total mass of all cell components including current collectors, Li anode, electrolyte, and separator. c) Cell specific energy for 70 wt% S_8_ and SP‐1N as a function of S loading at E/S ratio 7 µL mg^−1^. Redox (*C*_R_) contribution involving S and capacitive (*C*_c_) contributions from the carbon backbone for SP‐1N are also shown along with 35 wt% S_8_ (60% utilization). Energy is normalized by the total mass of all cell components. All cell level capacities are calculated at N/P ratio = 1.

At the electrode level, 70 wt% S_8_ clearly outperforms SP‐1N due to the excess cathode weight needed for SP‐1N to achieve the same S loading (**Figure** **9**a). The addition of capacitive contribution from carbon backbone is not sufficient to account for its excess mass at the electrode level. For example, S loading of 5 mg cm^−2^ in SP‐1N necessitates a total cathode material loading ≈20 mg cm^−2^ including additives and binders. On the other hand, a 70 wt% S_8_ cathode with S loading 5 mg cm^−2^ has a total cathode material loading of ≈7 mg cm^−2^ (Table S2, Supporting Information). A 1 cm^2^ electrode with 5 mg cm^−2^ S loading weighs ≈23 mg (including Al current collector) for SP‐1N compared to ≈10 mg for S_8_. This is reflected in the high *C*_el_ of S_8_ ≈510 mAh g_electrode_^−1^ compared to ≈280 mAh g_electrode_^−1^ for SP‐1N (Figure 9a). *C*_el_ for 70 wt% S_8_ dominates SP at all mass loadings as expected. Notwithstanding its poor performance at the electrode level, SP‐1N can match 70 wt% S_8_ (60% utilization) performance at low‐to‐moderate E/S (1–10 µL mg^−1^) and N/P ratios (1–10) at the cell level (*SE*_cell_ from Equation (6)) when all the inactive components are considered (Figure 9b and Table S2, Supporting Information). *SE*_cell_ for SP‐1N was close to 70 wt% S_8_ (Figure 9b) for all E/S ratios above 4 µL mg^−1^ (N/P = 1) due to the contribution (*C*_c_) from the carbon backbone. We demonstrate the effect of *C*_c_ using E/S ratio 7 µL mg^−1^ (Figure 9c). When *C*_c_ is not included in the calculation, SP‐1N cathodes yield *SE*_cell_ ≈60 Wh kg^−1^ for 5 mg cm^−2^ S loading at a E/S ratio of 7 µL mg^−1^ while a 35 wt% S_8_/C cathode (60% utilization) shows SE ≈155 Wh kg^−1^ (Figure 9c). *SE*_cell_ for SP‐1N increases to ≈185 Wh kg^−1^ at 7 µL mg^−1^ upon including *C*_c_, which is comparable to 70 wt% S_8_ cathode 60% utilization (≈175 Wh kg^−1^). From analyzing Figure 9b,c, it is evident SP‐1N with >5 mg cm^−2^ S loading can offset the inactive weight of other cell components at low to moderate E/S ratios (1–7 µL mg^−1^) similar to 70 wt% S_8_ despite the lower S content ≈35 wt%.

**Figure 10:**
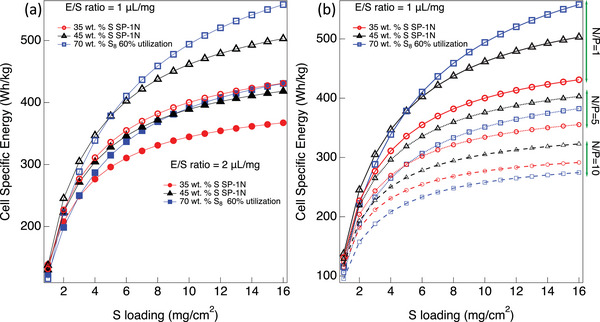
a) Cell specific energy for 70 wt% S_8_ and SP‐1N with 35 wt% and 45 wt% S at low E/S ratios as a function of S loading with N/P ratio = 1. Energy is normalized by the total mass of all cell components including current collectors, Li anode, electrolyte, and separator. c) Cell specific energy for 70 wt% S_8_ and SP‐1N with 35 wt% and 45 wt% S at different N/P ratios (1, 5, and 10) with E/S ratio = 1 µL mg^−1^.

At the cell level, a clear difference between S_8_ and SP‐1N is only visible for E/S <2 µL mg^−1^ and S loading >5 mg cm^−2^ (**Figure** **10**a). We also considered SP‐1N with ≈45 wt% S loading, which shows comparable cell level capacity with 70 wt% S_8_ at E/S = 2 µL mg^−1^ and N/P = 1. However, 70 wt% S_8_ shows better performance compared to 45 wt% SP‐1N at E/S = 1 µL mg^−1^ for S loading >8 mg cm^−2^. We varied the N/P ratio between 1 and 10 (Figure 10b). While 70 wt% S_8_ shows better capacity at N/P = 1, SP‐1N is found to perform better than 70 wt% S_8_ at higher N/P. The USABC proposes cell level energy ≈350 Wh kg^−1^ at C/3 discharge rate (equivalent to 235 Wh kg^−1^ at the pack level) as the goal for EVs.^[^ ^49^ ^]^ Based on this, we identified some critical metrics for SP‐1N materials with 3545 wt% S in **Table** **4**. For SP‐1N with 35 wt% S, we require S loading >5 mg cm^−2^ with E/S ratio <2 µL mg^−1^ and N/P ratio <5 for achieving 350 Wh kg^−1^. The required S loading is lower ≈4 mg cm^−2^ if SP‐1N S content is increased to 45 wt%. While higher S (>45 wt%) containing SP‐1N may seem better suited for LSBs, the presence of N in SP‐1N limits S doping to a maximum of <45 wt%. Furthermore, as discussed in Figure 4, higher S content compromises cycle stability due to the formation of longer S chains (e.g., SP‐2 and 3). We also evaluated the electrochemical performance of SP‐2 with 60 wt% S in different electrolytes (five mixed electrolytes) and three different additives (Fluoroethylene carbonate or FEC, LiNO_3_, and P_2_S_5_) to achieve the same stability as SP‐1 and SP‐1N with 35 wt% S. Although the initial specific capacity increased, there was no significant change in the rate of capacity fading (see Figures S6 and S7, Supporting Information) suggesting that SP at high S content behave similar to elemental S due to longer S chains. In summary, SP‐1N with 35 wt% content is still a viable alternative to S_8_ cathodes if a high S loading, low E/S, and N/P ratios can be achieved.

**Table 4:** Critical metrics for SP‐1N (with either 35 wt% or 45 wt% S content) for achieving 350 Wh kg^−1^ at the cell level with different E/S and N/P ratios

Cathode material	S‐loading [mg cm^−2^]	E/S [µL mg^−1^]	N/P
SP‐1N (45 wt% S)	4	1	1
SP‐1N (35 wt% S)	6	1	1
70 wt% S_8_	4	1	1
SP‐1N (45 wt% S)	7	2	1
SP‐1N (35 wt% S	11	2	1
70 wt% S_8_	7	2	1
SP‐1N (45 wt% S)	8	1	5
SP‐1N (35 wt% S	14	1	5
70 wt% S_8_	10	1	5

### 2.6 3D Current Collector for Achieving Critical Metrics

A major challenge for SP‐1N cathode preparation is the unviable thickness of SP‐1N at high S loadings >5 mg cm^−2^. As discussed above, *M*_cm_ for SP‐1N with ≈5 mg cm^−2^ S loading is more than twice that of 70 wt% S_8_. Although carbon backbone plays an active role that helps increase *SE*_cell_, SP‐1N electrodes are much thicker than S_8_ due to such excess weight. Furthermore, surface and bulk charge contributions do not scale linearly with thickness implying that *C*_c_ does not proportionately increase for thicker SP‐1N due to the inaccessibility of material embedded in deeper regions of thick electrodes. Thicker SP‐1N electrodes on Al/C current collector often develop cracks, delaminate with poor cycling stability, and exhibit high degree of electrochemical polarization. Indeed, we could not succeed in preparing SP‐1N electrodes with >2 mg cm^−2^ S loading on conventional Al/C foils (using wet slurry coating) as they either developed cracks or delaminated upon drying due to excess mass.

**Figure 11:**
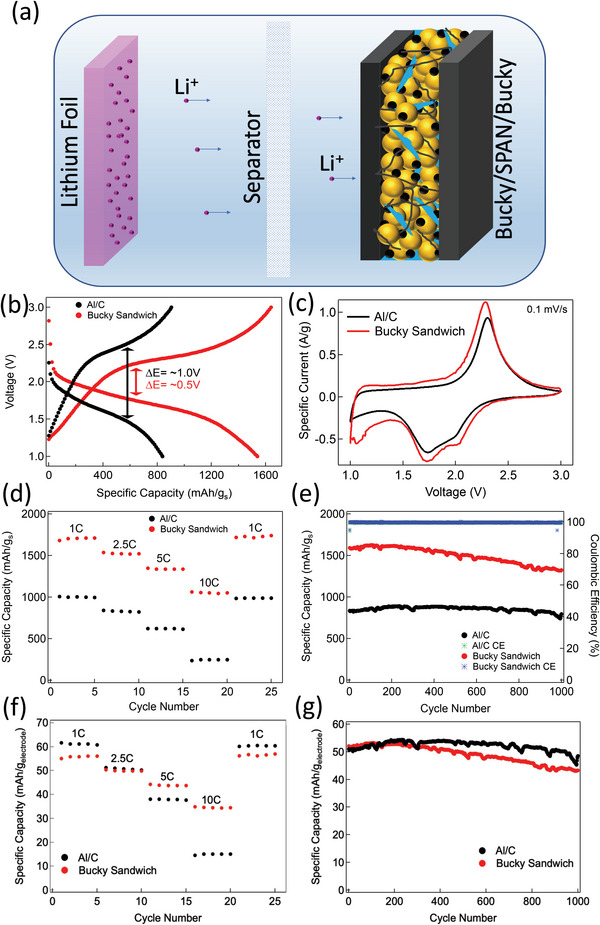
a) A schematic showing the BS SP‐1N sandwich structure. b) Charge and discharge profile of Al/C and BS electrodes (S loading: 0.4 mg cm^−2^) tested at 2.5C rate with corresponding polarization potential. c) Cyclic voltammograms of the Al/C and BS electrodes obtained at 0.1 mV s^−1^. d) The rate capability test of the electrodes at 1–10 C (1 C = 1675 mA g_s_^−1^). e) Cyclability of Al/C and BS electrodes tested at 2.5 C along with corresponding coulombic efficiency. Specific capacities are normalized by the sulfur mass. Electrode level normalization is presented in panels f) and g).

In order to surpass such limitations of Al/C foils, different metal, graphene, CNT foams, metal organic frameworks etc. have been used as 3D current collectors.^[^ ^50^, ^51^, ^52^, ^53^, ^54^, ^55^, ^56^, ^57^, ^58^, ^59^, ^60^, ^61^ ^]^ A detailed literature review of different SP electrodes is presented in Tables S3 and S4 in the Supporting Information. We previously used a 3D graphene foam (GF) current collector that allowed for loading ≈26 mg cm^−2^ S with SP‐1N, which corresponds to a total cathode material >106 mg cm^−2^. Despite the excellent performance of many 3D cathodes (e.g., GF) at the coin cell level, the practical manufacturing of pouch cells using 3D cathodes is often seriously limited by the fragility and the lack of roll‐to‐roll (R2R) processability of many 3D current collectors. We addressed these challenges using an interwoven CNT based BS structure (**Figure** **11**a) that can be processed using current R2R coating process similar to Al foil.^[^ ^62^ ^]^ We previously showed that Si nanoparticles embedded in BS outperform conventional Cu current collector.^[^ ^63^ ^]^ Building on this, we discuss the performance of SP‐1N in BS at both low (≈0.4 mg cm^−2^) and high (≈5.5 mg cm^−2^) S loadings.

### 2.7 Bucky Sandwich Performance at Low S Loading

The conventional Al/C current collector did not support high S loading. Accordingly, we first prepared a SP‐1N BS and Al/C samples with a low S loading of 0.4 mg cm^−2^ for comparing the CV and EIS of different current collectors (Figure 11b–e). As shown in Figure 11b, the gravimetric capacity of the SP‐1N BS was found to be superior to that of Al/C for all C rates at the S level. CV of SP‐1N BS did not show any significant difference in cathodic and anodic peak positions compared to SP‐1N on Al/C (Figure 11c) although the total area of the CV curve was found to be higher for SP‐1N BS. We tested the rate capability at different C‐rates: 1C, 2.5C, 5C, 10C, and 1C repeat after 10C (Figure 11d). At 1C the initial gravimetric capacity of SP‐1N BS was ≈1700 mAh g_s_^−1^, which is higher than the theoretical capacity of sulfur 1672 mAh g_s_^−1^ due to extra capacitive contribution arising from BS (see Figure S8a in the Supporting Information). On the other hand, SP‐1N on Al/C exhibited ≈1000 mAh g_s_^−1^. Despite the excess mass of BS, it performs as good as Al/C at the electrode level due to the extra capacitive contribution arising from the CNT structure (Figure S8a in the Supporting Information). We evaluated the cyclability 2.5C/2.5C charge/discharge rate for 1000 cycles (Figure 11e). After 1000 cycles, SP‐1N BS showed ≈81% capacity retention compared to Al/C electrodes with ≈94% capacity retention. Although it appears that capacity of SP‐1N BS is significantly better than Al/C at the sulfur level, the performance is indeed the same upon normalizing at the electrode level when the excess mass in BS is accounted (Figure 11f,g).

**Figure 12:**
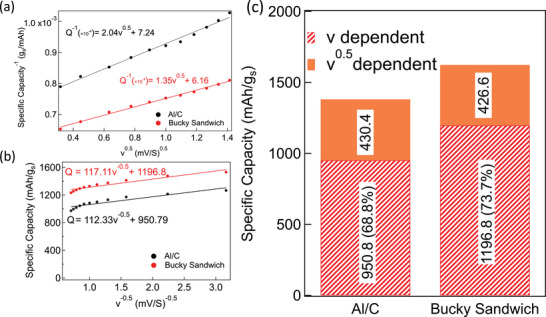
Scan‐rate (***v***) dependent cyclic voltammetry was performed using Trasatti's method (as discussed in Equations (3) and (4)) to obtain relationships between: a) the inverse of gravimetric capacity versus $\sqrt{v}$. The inverse of the *y*‐intercept (corresponding to infinitesimally slow scan rate) is the total charge and b) the gravimetric capacity versus $1/\sqrt{v}$. The *y*‐intercept of the liner fit (corresponding to infinitely fast scan rate) is the surface charge contribution. c) The deconvolution of surface (∝*v*) and bulk (∝$\sqrt{v})$contributions for SP‐1N on Al/C and BS current collectors.

As shown in **Figure** **12**a,b, we quantified the bulk and the surface contributions for SP‐1N BS electrodes similar to SP‐1N on Al/C using Equations (3) and (4). SP‐1N BS showed increased capacitive contribution ≈1197 mAh g_s_^−1^ due to additional surface area arising from CNTs within BS compared to ≈950 mAh g_s_^−1^ for Al/C (Figure 12c). The charge potential is often greater than the discharge potential due to polarization arising from the internal resistance (IR) of electrode materials. Such polarization decreases the discharge potential below the open circuit voltage while increasing the charge potential to reverse the chemical reaction at the electrode.^[^ ^64^ ^]^ The IR drop also leads to a drop in potential between the end of charge and the beginning of discharge. The difference between charge and discharge potentials at half specific capacity can be used to estimate the polarization potential. In case of SP‐1N BS, we found that polarization was smaller compared to Al/C at different C‐rates ranging from 0.1–10 C (Figure 11b and Figure S8b,c, Supporting Information).

### 2.8 EIS of SP‐1N Al/C and Bucky Sandwich Electrodes

**Figure 13:**
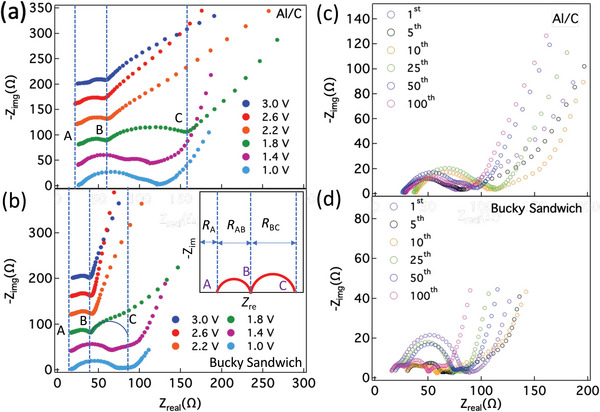
(a) and (b) show electrochemical impedance spectra (EIS) obtained during first cycle at various discharge voltages for Al/C and BS electrodes (S loading: 0.4 mg cm^−2^), respectively. The discharge was done at 0.1C rate. The inset in b) shows interpretation of electrode (*R*_A_), electrolyte (*R*_AB_), and mass/charge transfer (*R*_BC_) resistances based on semicircles in EIS spectra. Points A, B, and C for Al/C and BS are shown. A clear change is observed ≈1.8 V corresponding with CV and in situ Raman spectroscopy. In case of BS, the second semicircle overlaps with the nonvertical line (beyond point C due to capacitive contribution at lower frequencies). A semi‐circular fit is shown to identify point C. (c) and (d) show EIS spectra obtained at 1st, 5th, 10th, 25th, 50th, and 100th cycles for Al/C and BS electrode, respectively. The charge/discharge was done at 2.5C rate.

We analyzed EIS for both the first cycle and the first 100 cycles to identify electrode, electrolyte, charge transfer resistance (*R*_ct_) and capacitive features (**Figure** **13**). In EIS, an oscillating electric potential (*V*_ac_(*t*)) is imposed upon a constant potential (*V*_dc_) and the resulting current density *j*(*t*) is measured. These can be expressed as follows

(8) $$V\;\left(t\right)=V_{\mathrm{dc}}\;+V_{\mathrm{ac}}\left(t\right)\;=V_{\mathrm{dc}}\;+V_{o}e^{i\omega t}$$

(9) $$j\;\left(t\right)=j_{\mathrm{dc}}\;+j_{\mathrm{ac}}\left(t\right)\;=j_{\mathrm{dc}}\;+j_{o}e^{i\left(\omega t-\varphi \left(\omega \right)\right)}$$

In Equations (8) and (9), *V*_dc_ is the constant bias potential, *V*_o_ is the amplitude of the oscillating potential (typically <10 mV) at frequency *f* corresponding to *ω* = 2*πf*, *j*_dc_ is the timeindependent dc current density, *j*_o_ is the amplitude of the oscillating current density, and *φ*(*ω*) is the frequency‐dependent phase angle between *V*(*t*) and *j*(*t*). Then, the electrochemical impedance Z can be defined as

(10) $$Z\;\left(\omega \right)=\frac{V_{\mathrm{ac}}\left(\omega \right)}{j_{\mathrm{ac}}\left(\omega \right)}\;=Z_{\mathrm{re}}\;\left(\omega \right)+iZ_{\mathrm{im}}\left(\omega \right)$$

In Equation (10), *Z*_re_ and *Z*_im_ are the real and imaginary parts of the complex impedance, respectively.

Nyquist plots (Figure 13) present frequency‐dependence of impedance using *Z*_re_ and − *iZ*_im_ as the *x* and *y*‐axes. They typically consist of one or two semicircles at relatively high frequencies and a nonvertical line with respect to the real axis at low frequencies. There have been various interpretations of EIS data, which are often contradictory as discussed in ref. [65]. Mei et al. resolved these issues using detailed experimental and numerical analysis.^[^ ^65^ ^]^ They showed that the *x*‐axis (*Z*_re_) intercepts in the Nyquist plot, denoted as *R*_A_, *R*_AB_, and *R*_BC_, can be interpreted as the electrode resistance, the electrolyte resistance, and the sum of charge and mass transfer resistances, respectively (see the inset in Figure 13b).

During the first cycle, we found that *R*_A_ for Al/C and BS did not show any change. It is noted that *R*_A_ for Al/C is slightly greater than BS. In both cases, only one semicircle corresponding to *R*_AB_ was observed from 31.8 V. A second semicircle begins to appear ≈1.8 V along with a change in the slope of the nonvertical line, which is concomitant with the second cathodic peak (see the CV data in Figure 4 and Table 3). Below 1.8 V, the two semicircles overlap, which could be attributed to the fact that ion transport in the electrolyte and ion intercalation into the electrode (related to *R*_ct_) took place simultaneously throughout the electrode. Following the first cycle, we observed two semicircles and a nonvertical line up to 100 cycles with little variation in *R*_A_. *R*_A_ for BS remained slightly lower than Al/C even after 100 cycles. The nonvertical line beyond point C could be assigned to ion transport limitation in the electrolyte within or near the electrode. The slope of the line indicates whether the charging process is controlled by electric double layer (EDL) formation (large slope) or limited by ion diffusion (small slope). See the supporting information for a detailed discussion and mathematical derivation of the frequency dependence of the double layer formation and ion diffusion. The slope of the nonvertical line was higher for BS suggesting that charging occurs through EDL formation at low frequencies in the absence of Faradaic processes (Figure S9, Supporting Information). In case of Al/C, the nonvertical line arises from ion transport limitation within SP‐1N electrode. During the first cycle, BS showed more electric double layer features (lack of distinct second semicircle unlike Al/C at 1.8 V in Figure 13a,b) at lower frequencies similar to electric double layer capacitors. The slope of the nonvertical line did not significantly change between 10 and 100 cycles (Figure S9, Supporting Information). It is important to note that such a nonvertical line is not observed for planar redox active electrodes or Li‐ion batteries due to the dominance of Faradaic reactions at low frequencies (i.e., beyond point C).^[^ ^65^ ^]^ Overall, it is observed that the EIS features agree with our in situ Raman analysis and Trasatti plots showing that SP‐1N electrodes are closer to pseudo capacitors with both capacitive and redox contributions. The EIS for S_8_/C cathodes and SP‐1 are presented in Figures S10 and S11 in the Supporting Information.

### 2.9 Bucky Sandwich Performance at High S Loading

**Figure 14:**
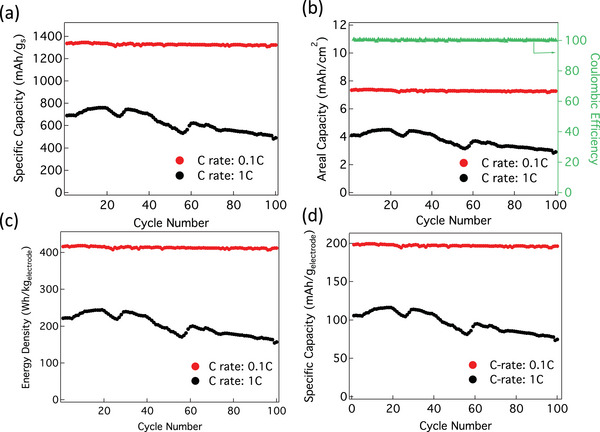
Cycling stability of the SP‐1N BS electrode (S loading ≈5.5 mg cm^−2^) in a coin cell configuration at 0.1C and 1C normalized by a) the mass of S, b) area, and c) electrode mass. d) Energy Density of the SP‐1N BS electrodes calculated using mass of the entire BS electrodes.

We were able to increase S loading to ≈5.5 mg cm^−2^ in BS (≈15.8 mg cm^−2^ of SP‐1N). As shown in **Figure** **14**, we evaluated the electrochemical performance and rate capability of SP‐1N BS electrode with ≈5.5 mg cm^−2^ S‐loading at a moderate E/S ratio ≈7 µL mg^−1^ in line with critical metrics derived for SP‐1N in Figures 9 and 10.

**Figure 15:**
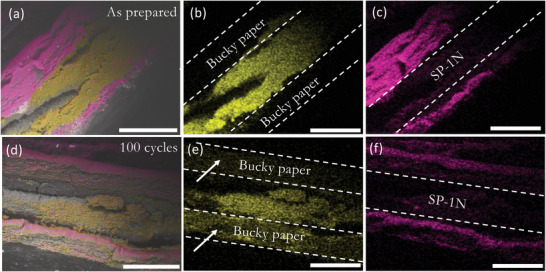
a)–(c) show false‐color elemental maps for as prepared SP‐1N bucky sandwich structure. While (a) shows a composite map combining S and C maps, (b) and (c) show maps for S and C. S was mainly present within the sandwich with no S in bucky paper. (d)–(f) show composite, S, and C maps for the electrode after 100 cycles. The arrows in (e) show that S is diffused into bucky papers after 100 cycles. Dashed lines in (b), (c), (e), and (f) are provided as a guide to the eye. All scale bars are 500 µm.

**Figure 16:**
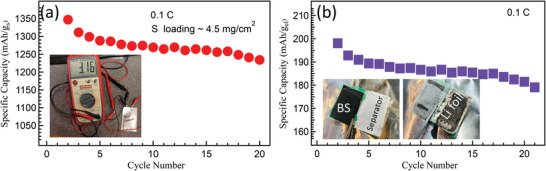
Cycling stability of the SP‐1N BS electrode (S loading ≈4.5 mg cm^−2^) in a pouch cell configuration at 0.1C normalized by the total mass of a) S and b) electrode. The inset in (a) shows a photograph of BS pouch cell with 3.16 V voltage. Although the BS structure remained intact after 100 cycles (see insets in (b)), the cyclability was compromised my deterioration of Li foil.

The cycling capability test was performed at 0.1C and 1C rate, which showed a gravimetric capacity ≈1360 mAh g_s_^−1^ (≈200 mAh g_el_^−1^) and ≈690 mAh g_s_^−1^ (≈100 mAh g_el_^−1^) respectively. The areal capacity is ≈7.8 mAh cm^−2^ and ≈4.0 mAh cm^−2^ at 0.1C and 1C rates, which is higher than that the current LIB's and comparable with the values found in literatures (see Table S4, Supporting Information). We used elemental mapping in an electron microscope to evaluate the structural integrity of SP‐1N BS after 100 cycles as shown in **Figure** **15**. Clearly, SP‐1N is mainly present only within the sandwich structure before cycling (Figure 15a–c). After 100 cycles, we observed that some S diffuses into CNT BS (Figure 15d–f) while retaining the structural integrity. We also assembled pouch cells using high S loading BS cathode (Figure 15). Our pouch cells showed ≈200 mAh g_el_^−1^ at the electrode level at 0.1 C. The cyclability of our pouch cell was limited by commonly observed degradation of Li foil rather than the cathode (see Figure S12, Supporting Information) (**Figure** **16**).

## 3 Conclusions

In summary, we showed that SP cathodes are stable at low S content (≈35 wt%) with or without N atoms due to predominantly shorter S chains. SP materials with higher S content contain a larger proportion of longer S chains, which lead to rapid capacity degradation. The carbon backbone in SP‐1N contributes significantly to the overall capacity. In the absence of N (SP‐1), low DOS(*E*_F_) results in low *Q*_C_, which limits total capacity mainly to redox contribution. Overall, our in situ Raman, scan rate dependence measurements, and EIS show that SP‐1N behaves like a pseudocapacitor meaning that SP‐1N with 35 wt% S cannot be treated on the same footing as S_8_/C with 35 wt% S due to the active nature of the carbon backbone. In light of this, we derived critical metrics for SP‐1N by considering capacitive contributions. Although SP‐1N shows poor performance compared to S_8_/C (70 wt% S with 60% utilization) at the electrode level, we demonstrated that it matches S_8_/C performance at the cell level for S loading >5 mg cm^−2^ (>15 mg cm^−2^ of SP‐1N) and E/S ratios 2–10 µL mg^−1^. However, SP‐1N cathodes are thicker than S_8_/C for the same S loading, which imposes challenges with ion diffusion, delamination, and polarization when used with flat Al/C current collectors. Using CNT‐based BS current collectors, which are amenable to R2R production similar to Al/C, we were able to achieve higher S loading (5.5 mg cm^−2^). The extra capacitive contribution from BS structures compensates for its excess mass without compromising the total capacity at the electrode level. The cycling capability test of SP‐1N BS at high loadings showed a gravimetric capacity ≈1360 mAh g_s_^−1^ (≈200 mAh g_el_^−1^) at 0.1C and ≈690 mAh g_s_^−1^ (≈100 mAh g_el_^−1^) at 1C. We also prepared SP‐1N BS pouch cell, which showed ≈1300 mAh g_s_^−1^ (≈190 mAh g_el_^−1^) at 0.1C, to demonstrate the practical applicability of SP material based Li—S pseudocapacitors.

## 4 Experimental Section

### Chemicals and Materials

Poly(vinylidene fluoride) (PVDF), lithium bis(trifluoromethane) sulfonamide (LiTFSI), lithium hexafluoro phosphate (LiPF_6_), ethylene carbonate (EC), propylene carbonate, 1,2‐dimethoxyethane (DME), 1,3‐dioxolane (DOL), dimethyl carbonate, ethyl methyl carbonate, *N*‐methyl‐2‐pyrrolidone (NMP), potassium hydroxide (KOH), PAN and PPS were purchased from Sigma‐Aldrich. The sulfur (S) powder (325 mesh) was purchased from Alfa Aesar. Carbon super P, lithium chips/foil, and carbon coated Al‐foil (Thickness 18 µm) were purchased from MTI Corporation (mtixtl.com) Bucky papers (60 gsm) were obtained from Nanotech Labs, Yadkinville, NC.

### SP Synthesis

For preparing SP samples without N atoms (labeled SP‐1, SP‐2, and SP‐3), 2 g of PPS and 4 g of KOH were dissolved in the 50 ml of water and ethanol solution (7:3 ratio) using magnetic stirrer for 2 h and then the solution was dried at 110 °C for 48 h in ambient conditions. The obtained dried powders were mixed with 2, 6, and 12 g of elemental sulfur for preparing SP‐1, 2, and 3 samples respectively. Then the mixture was annealed at 650 °C for 2 h in a ½″ quartz tube at a ramp rate of 5 °C min^−1^ in N_2_ atmosphere. Obtained samples were ball milled for 20 min. Sulfurized polymer with N atoms (labeled SP‐1N) was prepared using PAN and S. PAN and S were mixed in 1:3 ratio and annealed in N_2_ atmosphere for six hours at 450 °C in a ½″ quartz tube.

### Coin/Pouch Cell Preparation and Electrochemical Testing

Slurry was prepared by mixing SP samples with PVDF and Super P (7:1.5:1.5 ratio) in NMP. The as prepared slurry was coated on carbon coated Aluminum and bucky paper using Doctor blade with a vacuum bed (MTI corp). In case of BS electrodes, another noncoated bucky paper was added on top of coated piece before drying. The coated electrodes were air dried for 12 h followed by oven dry at 110 °C for another 12 h. 10 mm coupons were punched out and used as the electrode for CR2032 type coin cells. For the pouch cells, 2×3 cm electrodes were used. A Li chip (15.6 mm diameter × 0.45 mm thickness)/Lithium coated copper foil (Li‐ thickness 100 µm/ and 2.5 cm*3.0 cm) was used as the counter electrode for coin/pouch cells, respectively, Celgard 2325 was used as the separator and 1 m LiTFSI (lithium bis(trifluoromethanesulfonyl)imide) in EC_0.5_DME_0.25_DOL_0.25_ (EC, DME, and DOL) was used as the electrolyte. All samples were evaluated in four more electrolytes discussed in the supporting information. For lower sulfur loadings (0.4 mg cm^−2^), the amount of electrolyte was fixed to 35 µL, whereas for higher loadings (>4 mg cm^−2^) E/S ratio of 7 µL mg^−1^ was used. The galvanostatic charge/discharge was performed using MTI and Arbin battery analyzer system in the voltage range of 1 to 3 V. Electrochemical impedance spectroscopies (from 1 MHz to 0.1 Hz by applying ac amplitude of 10 mV) and cyclic voltammetry (from 1 to 3 V) was measured using Gamry 3000 potentiostant. Micro‐Raman spectroscopy was performed using Renishaw InVia Raman microscope couple with 532 nm excitation. To obtain the real‐time spectra during charge/discharge, the acquisition time was limited to 10 s per spectrum with two accumulations.

### Other Characterization

Scanning electron microscopy was performed using Hitachi SEM4800. Thermogravimetry was obtained using universal V4.5A TA instruments. Carbon, Hydrogen, Nitrogen, Sulfur, and Oxygen (CHNSO) elemental analysis was performed by Atlantic Microlabs (Norcross, GA). X‐ray photoemission spectra were obtained with Kratos Axis Supra XPS (X‐ray source: monochromated Al *K*_*α*_, multichannel plate, and delay line detector with a take‐off angle of 90°). Autosorb iQ was used to measure the surface area of the graphene foam and the cathode electrode.

## Conflict of Interest

The authors declare no conflict of interest.

## Author Contributions

N.S. and S.C. contributed equally to this work. R.P. instigated and designed the project. P.P. performed the initial literature review, synthesis, and characterization in collaboration with N.S. and S.C. N.S. and S.C. led spectroscopic, microscopic, and electrochemical characterization under the guidance of R.P. R.P. and A.R. performed critical metric analysis. The paper was drafted by R.P., N.S., and S.C. All the authors discussed the paper.

## Supporting information

Supporting Information

## Data Availability

The data that support the findings of this study are available from the corresponding author upon reasonable request.
