# Supplementary material for: Insights into the Pseudocapacitive Behavior of Sulfurized Polymer Electrodes for Li–S Batteries
Source: Adv Sci (Weinh). 2023 Mar 30;10(15):2206901. doi: 10.1002/advs.202206901 (PMC10214234; doi:10.1002/advs.202206901)
Supplement: Supplementary file 1 — Supporting Information [file ADVS-10-2206901-s001.pdf]

**Supporting Information**

# Insights into the pseudocapacitive behavior and industrial translation of sulfurized polymer electrodes for Li-S batteries

Nawraj Sapkota, Shailendra Chiluwal, Prakash Parajuli, Alan Rowland, and Ramakrishna Podila*

## Experimental Section:

**Mixed electrolytes**: We used two traditional electrolytes (TE): 1) **TE-I**: 1M LiTFSI in DME:DOL = 1:1 and 2) **TE-II**: 1M $LiPF_6$ in EC:DMC:EMC = 1:1:1. In addition, we also tested some cells in three new electrolytes: 3) **NE-I**: 1M $Li(PF_6)_{0.3}(TFSI)_{0.7}$ in $EC_{0.1}$:$DMC_{0.1}$:$EMC_{0.1}$:$DME_{0.35}$:$DOL_{0.35}$ 4) **NE-II**: 1M LiTFSI in $EC_{0.5}DME_{0.25}DOL_{0.25}$, and 5) **NE-III**: 1M LiTFSI in $PC_{0.8}DME_{0.1}DOL_{0.1}$. All ratios are volume based. The solvent abbreviations are as follows: EC: ethylene carbonate, PC: propylene carbonate, EMC: ethyl methyl carbonate, DMC: dimethyl carbonate, DME: 1,2-dimethoxyethane and DOL: 1,3-dioxolane. We also used the following additives for SP-2 to test their effectiveness in reducing the capacity fade: 1 and 5 wt. % fluoroethylene carbonate (FEC), 0.3M $LiNO_3$, and 0.1M $P_2S_5$.

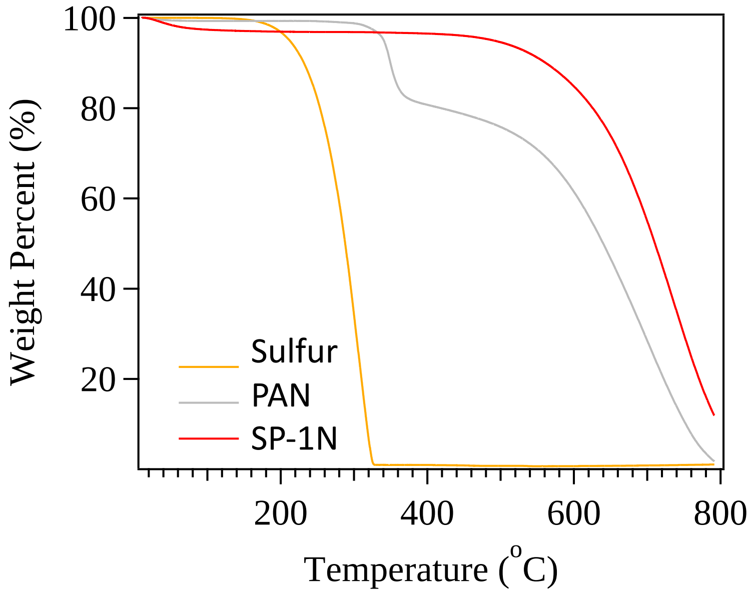


**Figure S1**: Thermogravimetric analysis of elemental sulfur, PAN and SP-1N.

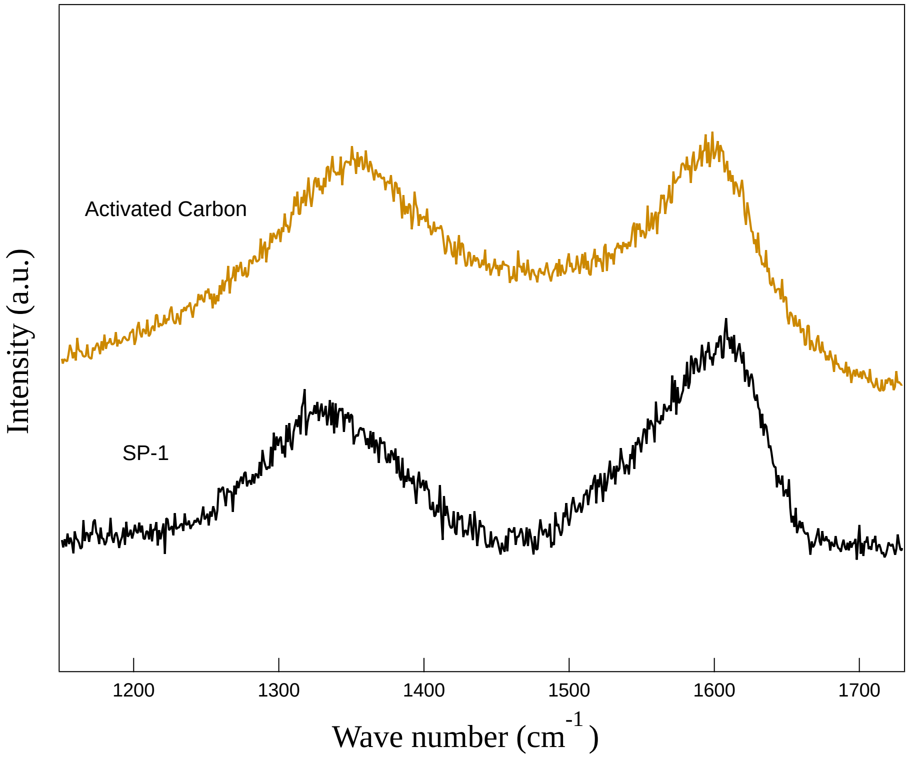


**Figure S2**: A comparison of the Raman spectra of activated carbon and SP-1 in 1200-1700 cm$^{-1}$ shows similarity in their carbon bonding environment.

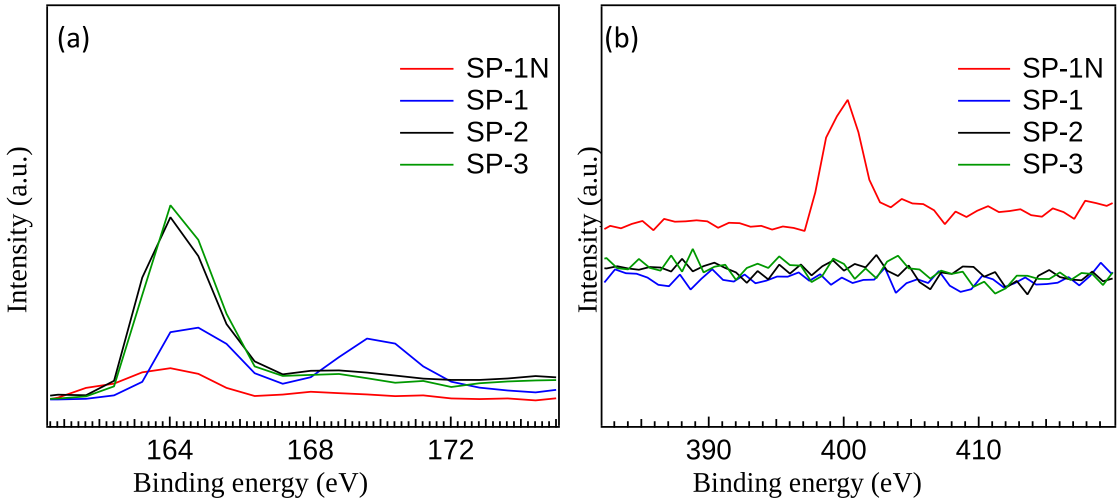


**Figure S3**: X-ray photoemission spectra of all SP samples showing (a) S and (b) N peaks. Only SP-1N contains N atoms in non-graphitic configuration.

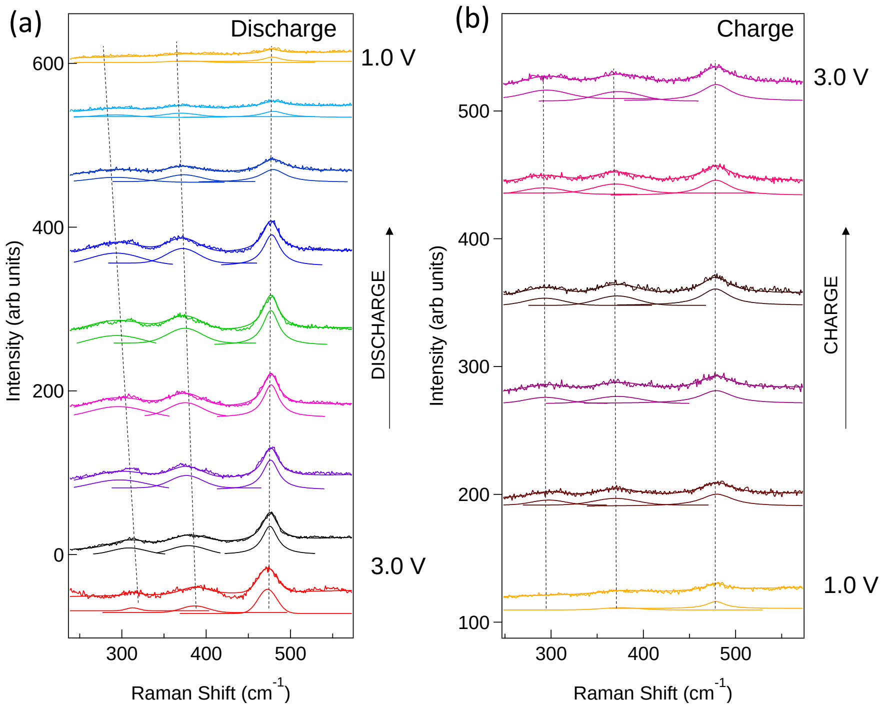


**Figure S4**: In-situ Raman spectroscopy of SP-1N during the first cycle (a) discharge from 3.0 to 1.0 V and (b) charge from 1.0 to 3.0 V. All the spectra were fit using Lorentzian peaks. Dashed lines are provided as a guide to the eye to observe spectral shifts.

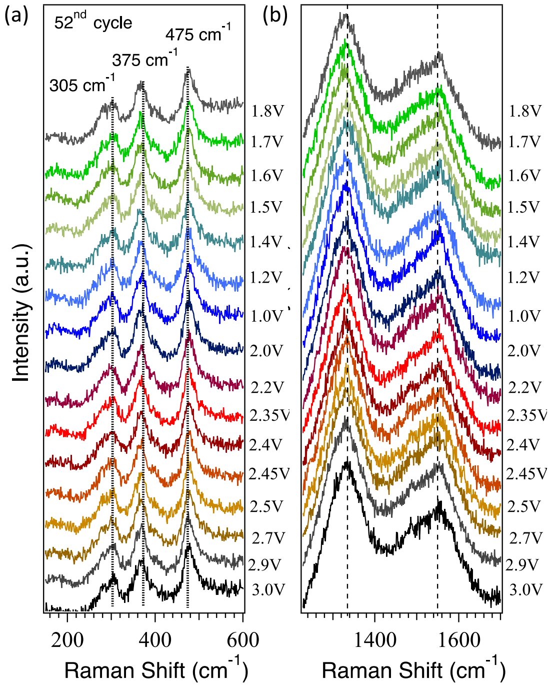


**Figure S5**: (a) and (b) show in-situ Raman spectra for SP-1N cathode in 52nd cycle.

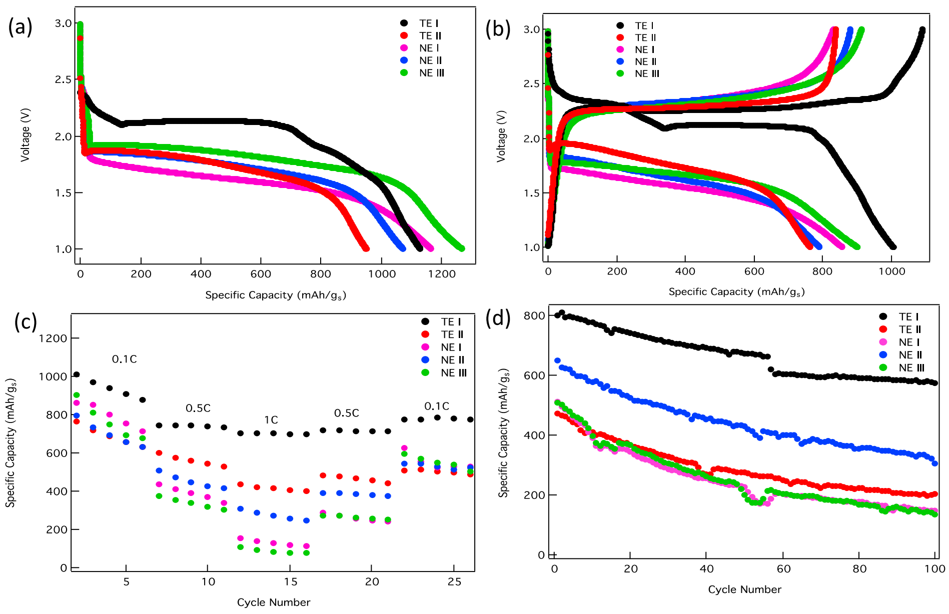


**Figure S6**: Investigation of the effect of different electrolytes on the performance of SP-2 with 60 wt. % S showing (a) first discharge capacity, (b) charge discharge plot, (c) rate capability and (d) cycling stability. While some electrolytes increase the initial capacity (e.g., TE-1) relative to NE-II, other electrolytes showed a decrease in capacity (TE-II, NE-I, and NE-III). However, SP-2 showed similar capacity fading in all electrolytes. The S loading is 0.4 mg/cm$^2$.

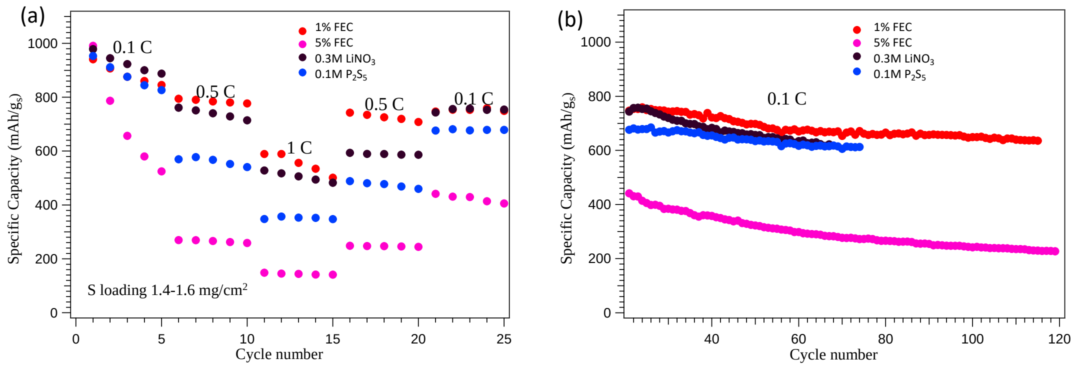


**Figure S7**: Investigation of the effect of different additives on the performance of SP-2 with 60 wt. % S loading in NE-II electrolytes (a) rate capability and (b) cycling stability. While 1% FEC, $LiNO_3$ and $P_2S_5$ showed some improvement in capacity fading, the capacity decreases significantly from 780 to 680 mAh/g$_S$.

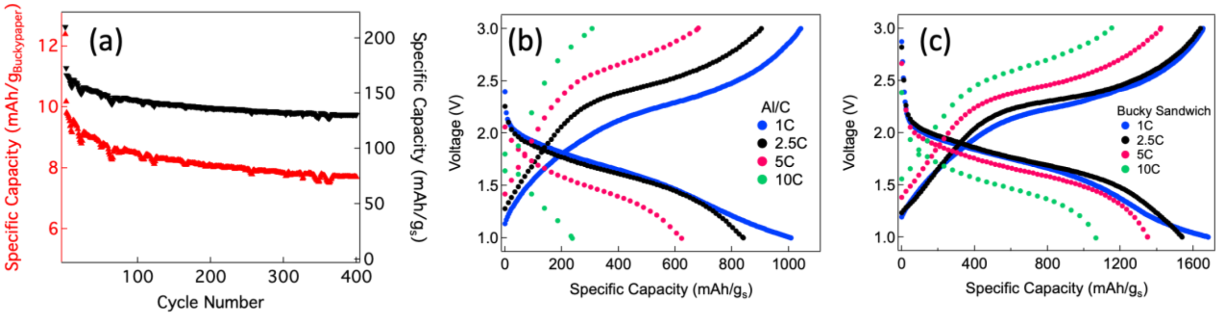


**Figure S8:** (a) Specific capacity of the bucky paper tested at 0.1C rate (1C = 1675 mAh/g) without slurry. Left and right axis are two mass normalization condition one is using mass of the Bucky paper and another is using mass of sulfur, (b) and (c) a typical charge and discharge curve for Al/C and bucky sandwich electrodes at different c-rate respectively. The S loading in (b) and (c) is 0.4 mg/cm$^2$.

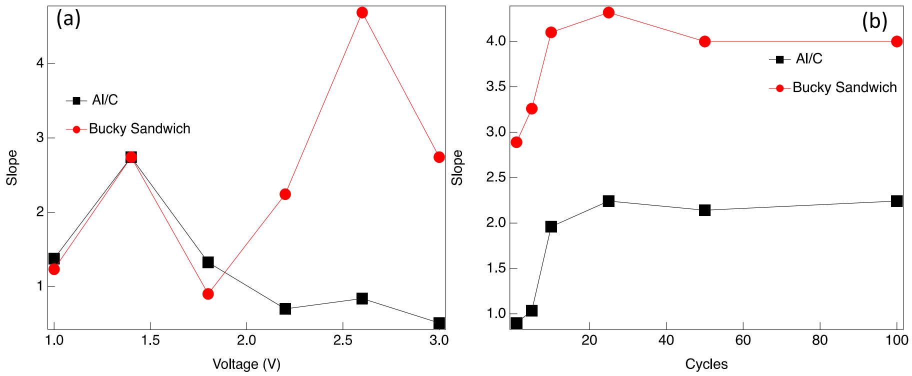


**Figure S9**: The slope of non-vertical line (beyond point C in Fig. 13) for SP-1N on Al/C and buckysandwich for (a) first discharge and (b) for the first 100 cycles.

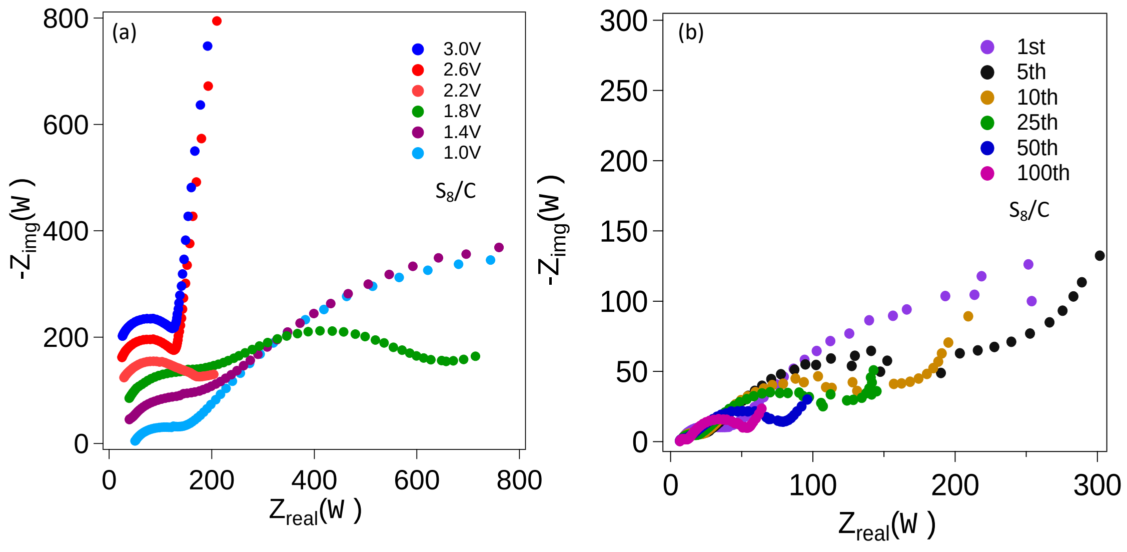


**Figure S10**: (a) shows electrochemical impedance spectra (EIS) obtained during first cycle at various discharge voltages for $S_8$/C coated on Al/C (S loading: ~1 mg/cm$^2$) at a discharge rate of 0.1C. (b) shows EIS spectra obtained at 1$^{st}$, 5$^{th}$, 10$^{th}$, 25$^{th}$, 50$^{th}$ and 100$^{th}$ cycles $S_8$/C coated on Al/C at a discharge rate of 2.5C.

As shown in Fig. S10a, we found that $S_8$/C electrodes initially showed double layer capacitance up to 2.6 V. We attribute this to ~30 wt. % SuperP conducting C that was added to make $S_8$/C electrode electrically conducting. Upon the initiation of lithium sulfide formation below 2.6 V, the capacitive feature disappears and broad circular features corresponding to the formation of various higher and lower Li polysulfides are observed. At 1.8 V, $Li_2S$ formation occurs with almost no capacitive features. The evolution of EIS spectra for $S_8$/C samples over 100 cycles is shown in Fig. S10b.

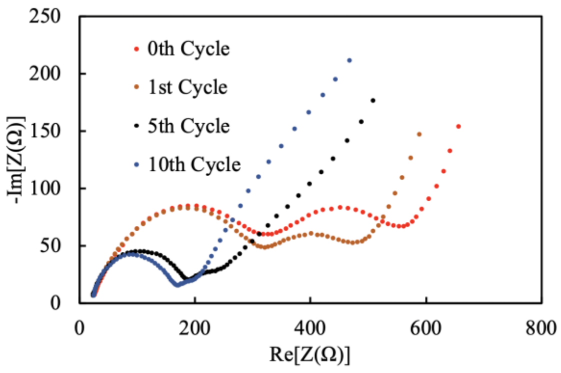


**Figure S11**: Electrochemical impedance spectra for SP-1 samples in the first 10 cycles show that any S-O bonds are plausibly broken upon the formation of $Li_2S$ leading to a reduction in low frequency redox process (indicated by semi-circular features).

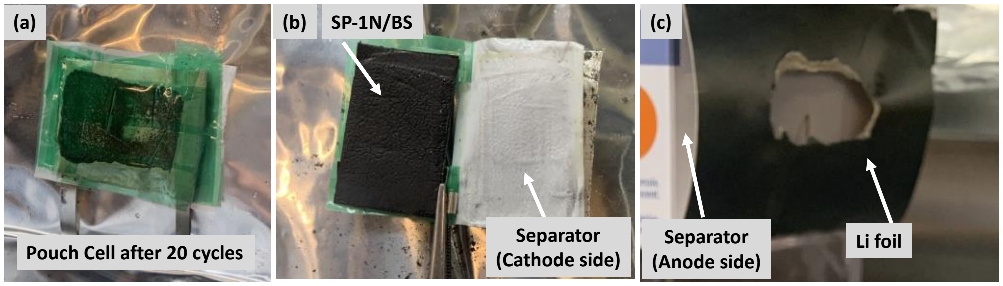


**Figure S12**: (a-c) Photographs of pouch cell assembled using SP-1N and bucky sandwich (BS) structure (SP-1N/BS) and Li metal foil after 20 cycles. Upon opening the pouch cell inside the glove box, SP-1N/BS and the separator (on the cathode side) were found to be in a state similar to their original condition (b). However, as shown in (c), we Li foil accumulated significant debris and deteriorated leading to cell failure.

**Double-layer and ion-diffusion processes in EIS**: In case of ideal electric double layer formation, there is no charge transfer or depletion of redox active species concentration near the electrode. Considering the capacitance to be constant, the charge is proportional to the potential (*V*).

$$V(t) = \frac{Q(t)}{C_{dl}} \quad (1).$$

From the above equation, the rate of change of charge (i.e., current) is seen to be proportional to the rate of change of potential.

$$\frac{dV(t)}{dt} = \frac{I(t)}{C_{dl}} \quad (2).$$

Upon performing a Fourier transform on equation 2, we can see that

$$i\omega\hat{V}(\omega) = \frac{\hat{I}(\omega)}{C_{dl}} \quad (3).$$

$$Z(\omega) = \frac{\hat{V}(\omega)}{\hat{I}(\omega)} = \frac{1}{i\omega C_{dl}} \quad (4).$$

Thus, the Nyquist plot for an ideal double layer has no real component. If a sine wave potential is applied, the resulting current is a cosine, and a phase shift of $-\frac{\pi}{2}$ (corresponding to a vertical line in the Nyquist plot) is observed. Although $C_{\mathrm{dl}}$ is ideally independent of frequency, the experimentally measured phase shift depends on frequency due to additional circuit elements in series and in parallel arising from surface roughness, chemical heterogeneity, nonuniform charge adsorption etc. Thus, lines with high slope in the Nyquist plot are related to $C_{\mathrm{dl}}$.

On the other hand, if a redox process is present, some redox species (e.g., $Li^+$ ions) are consumed leading to the concentration gradient near the electrode surface. If the charge consumed in the redox process is $\Delta Q_{red}$ (a fraction of the concentration of redox species present near the electrode surface) for a given change in potential ($\Delta V$), we may express the diffusion capacitance ($C_{diff}$) as

$$C_{diff} = \frac{\Delta Q_{red}}{\Delta V} \quad (5).$$

Suppose that concentration of depleted ions is given by $\Delta c$ within a distance $d$ from the electrode surface, we may express

$$\Delta Q_{red} = nFd\Delta c \quad (6).$$

Considering $D$ to be the diffusion coefficient of ions, it may be seen that $d$ (average region of ion depletion) is dependent on frequency as

$$d = \sqrt{\frac{D}{\omega}} \quad (7).$$

Therefore, we find that $C_{diff} = \frac{nF\Delta c}{\Delta V}\sqrt{\frac{D}{\omega}}$ is dependent upon frequency. We can re-write this as

$$C_{diff} = \sigma^{-1}\omega^{-1/2} \quad (8).$$

We define $\sigma^{-1} = \frac{nF\Delta c}{\Delta V}\sqrt{D}$.

Given that $\Delta Q_{red} = nFd\Delta c = nF\Delta c\sqrt{\frac{D}{\omega}}$, the diffusion current is

$$\Delta I_{diff} = nF\Delta c\sqrt{D\omega} \quad (9).$$

This corresponds to a real resistance for charge flow

$$R_{diff} = \frac{\Delta V}{nF\Delta c\sqrt{D\omega}} = \sigma\omega^{-1/2} \quad (10).$$

We can express the total impedance as

$$Z(\omega) = \sigma\omega^{-1/2} - i\sigma\omega^{-1/2} \quad (11).$$

As it can be seen, $Z_{re}(\omega)$ and $Z_{Im}(\omega)$ both have the same frequency dependence, which leads to a $\frac{\pi}{4}$ phase shift which has lower slope than double layer formation.

Please note this is a heuristic derivation. A fully rigorous derivation could be found in J. Randles, Discuss. Faraday Soc., 1947,1, 11-19.

**Table S1**: List of parameters and values used for calculating theoretical cell level performance of Li-S with elemental S and SP-1N.

| **Parameter** | **Value** |
|---|---|
| Li metal density | 0.534 g/cc |
| $S_8$ density | 2.07 g/cc |
| Carbon density | 2.2 g/cc |
| Binder density | 1.76 g/cc |
| Electrolyte density | 1.1 g/cc |
| Al current collector density | 2.7 g/cc |
| Bucky paper areal weight | 60 g/m$^2$ |
| Cu current collector density | 8.9 g/cc |
| Separator density | 0.57 g/cc |
| Al foil thickness | 10 mm |
| Nominal cell voltage | 2.1 V |
| Cu foil thickness | 10 mm |
| N/P ratio | 1 |
| S loading | Varied from 2-16 mg/cm$^2$ |
| E/S ratio | Varied from 1-10 mL/mg |
| S theoretical capacity | 1672 mAh/g |
| Li theoretical capacity | 3680 mAh/g |

**Table S2**: A snapshot of different variables in equations

| Sample | S loading (mg/cm$^2$) | $M_{CM}$ (mg) | $M_{ca}$ (mg) | $M_{Li}$ (mg) | $M_{An}$ (mg) | E/S ratio | $M_{El}$ (mg) | $M_{Total}$ (mg) | $C_{total}$ (mAh) | $C_S$ (mAh/$g_s$) | $C_{el}$ (mAh/$g_{el}$) | $SE_{Cell}$ (Wh/kg) |
|---|---|---|---|---|---|---|---|---|---|---|---|---|
| SP-1N | 5 | 20.5 | 23.5 | 1.7 | 11.7 | 2 | 2.2 | 46 | 6.5 | 1300 | 276 | 290 |
| 70% $S_8$ 60% Utilization | 5 | 7 | 10 | 2.2 | 12.2 | 2 | 2.2 | 33 | 5 | 1003 | 500 | 318 |

**Table S3:** Various methods of SPAN synthesis along with the two-dimensional current collector used, maximum S-loading achieved and electrochemical performance.
A: Sample name, B: SPAN synthesis method, C: S content in SPAN (%), D: Active material: binder: carbon ratio in slurry, E: Current collector, F: S loading in electrode (mg cm$^{-2}$), G: Electrochemical performance, H: Year of the publication and reference**.**

| A | B | C | D | E | F | G | H |
|---|---|---|---|---|---|---|---|
| S/DPAN | S: PAN = 4:1, Temp: 300 °C | 48% | 8:1:1 | Ni foam | 2.69 | 1050 mAh g$^{-1}$ after 80 cycles at 0.2C | 2014[1] |
| Porous PAN/S | S & PAN/PMMA nanofiber, Temp: 300°C | 48% | 7:2:1 | Al foil | NA | 1144 mA h g$^{-1}$ after 100 cycles at 0.2 C | 2021[2] |
| SPAN 4 | S: PAN = 4:1 Temp: 450 °C | 45.6% | 7:1.5:1.5 | C coated Al foil | 0.39 | >1000 mA h g$^{-1}$ after 100 cycles at 0.4C | 2015[3] |
| SPAN | S: PAN Temp: 350°C | 32% | 7:1.5:1.5 | Al foil | 0.22 | 938 mA h g$^{-1}$ after 450 cycles at 0.9C rate | 2017[4] |
| MSPAN | S: SBA-PAN = 8:1, Temp: 155°C, 330°C & 550°C | 45.9% | 8:1:1 | Al foil | 1.12 | 755 mAh g$^{-1}$ after 200 cycles at 1 C | 2017[5] |
| S/MPCPs-PAN | S: MCP-PAN = 6:1, Temp: 155°C, 300°C | 54% | 8:1:1 | Al foil | 1.0 | 789.7 mAh g$^{-1}_{composite}$ after 200 cycles at 160 mA g$^{-1}_{sulfur}$ | 2017[6] |
| BP-SPAN- | S: PAN: BP | 43% | 8:1:1 | C | 1.0 | 1266 mA h g$^{-1}$ after100 | 2020[7] |

| | | | | | | | |
|---|---|---|---|---|---|---|---|
| 2 | Temp: 300°C | | | coated Al foil | | cycles at 0.1C | |
| DH-150-300-SPAN | S: PAN = 4:1, Temp: 155°C, 300°C | 38.3% | 8:1:1 | Ni foam | 3.0 | 1000 mAh $g^{-1}$ after 150 cycles at 0.1 C | 2020[8] |
| S@pPAN | S: PAN = 3:1, Temp: 300°C | 37.6% | 8:1:1 | Al foil | 2.5 | 1702 mAh $g^{-1}$ after 100 cycles at 0.1 C | 2018[9] |
| SPAN | S & PAN Temp: 330°C | 39.8% | 8:1.4:0.6 | Al foil | 0.6 | 1380 mAh $g^{-1}$ after 2000 cycles at 500 mA/g | 2018[10] |

**Table S4:** Comparison of various materials used in LSBs, current collector, sulfur loading, E/S ratio, electrochemical performance found in recently published papers.

| Material Preparation | Current Collector | S loading | E/S ratio (µL/mg)/ Amount of electrolyte (µL) | Electrochemical Performance | Year/ Ref. |
|---|---|---|---|---|---|
| HPCFs/ S | Al foil | 1.15 mg | 150 µL/mg | 754 mAh/g after 500 cycles at 0.2C | 2021[11] |
| CNT Dry film- S | Film itself | 2.0 mg $cm^{-2}$ | 7.0 µL/mg | 828 mAh/g after 90 cycles at 0.1C | 2021[12] |
| GF-SPAN | Graphene foam | 26.5 mg $cm^{-2}$ | 7.0 µL/mg | 19.2 mAh/$cm^2$ after 50 cycles at 3.3 mA/g | 2021[13] |
| c-(rGO-$CoS_2$)/S film | Film itself | 5.8 mg $cm^{-2}$<br>7.2 mg $cm^{-2}$<br>8.3 mg $cm^{-2}$ | 5.0 µL/mg | 4.1 mAh/$cm^2$<br>4.86 mAh/$cm^2$<br>5.5 mAh/$cm^2$ after 50 cycles at 0.1C | 2021[14] |
| S/$LiNi_{0.8}Co_{0.1}Mn_{0.1}O_2$ | Al foil | 4.29 mg $cm^{-2}$ | 10.0 µL/mg | 600 mAh/g after 120 cycles at 0.1C | 2020[15] |
| PGCNF/S aerogel | Material itself | 15.8 mg $cm^{-2}$ | 135 µL | 1028 mAh/g after 600 cycles at 0.5C | 2020[16] |
| Mesoporous TiN/S | Carbon coated Al | 0.59 mg $cm^{-2}$ | NA | 644 mAh/g after 500 cycles at 0.5C | 2016[17] |

| | | | | | |
|---|---|---|---|---|---|
| Sulfur-3D CNT foam | Material itself | 19.1 mg $cm^{-2}$ | NA | 500 mAh/g (9 mAh $cm^{-2}$) after 100 cycles at 0.1C | 2017[18] |
| NDHC@C–S | Al foil | 4.0 mg $cm^{-2}$ | 12.0 µL/mg | 400 mAh/g after 500 cycles at 0.5C | 2020[19] |
| BTO-CNT-S | Al foil | 2.5 mg $cm^{-2}$ | NA | 588 mAh/g after 100 cycles at 1C | 2020[20] |
| NMRC/S@$MnO_2$ | Al foil | 1.8 mg $cm^{-2}$ | 15.0 µL/mg | 590 mAh/g after 1000 cycles at 2C | 2020[21] |
| Bucky/SPAN/Bucky | Bucky paper | 0.4 mg $cm^{-2}$ | 35 µL | 1300 mAh/g after 1000 cycles at 2.5C | This work |
| Bucky/SPAN/Bucky | Bucky paper | 5.5 mg $cm^{-2}$ | 7.0 µL/mg | 1360 mAh/g after 100 cycles at 0.1C | This work |
| Bucky/SPAN/Bucky | Bucky paper | 5.5 mg $cm^{-2}$ | 7.0 µL/mg | 690 mAh/g after 100 cycles at 1C | This work |

References:


[1] A. Konarov, D. Gosselink, T. N. L. Doan, Y. Zhang, Y. Zhao, P. Chen, *Journal of Power Sources* **2014**, *259*, 183.

[2] K. Wang, S. Ju, Q. Gao, G. Xia, G. Wang, H. Yan, L. Dong, Z. Yang, X. Yu, *J Alloys Compd* **2021**, *860*, DOI 10.1016/J.JALLCOM.2020.158445.

[3] S. Wei, L. Ma, K. E. Hendrickson, Z. Tu, L. A. Archer, *Journal of the American Chemical Society* **2015**, *137*, 12143.

[4] Y. Li, Q. (Ray) Zeng, I. R. Gentle, D.-W. Wang, *Journal of Materials Chemistry A* **2017**, *5*, 5460.

[5] Y. Liu, A. K. Haridas, K.-K. Cho, Y. Lee, J.-H. Ahn, *The Journal of Physical Chemistry C* **2017**, *121*, 26172.

[6] Y.-Z. Zhang, Z.-Z. Wu, G.-L. Pan, S. Liu, X.-P. Gao, *ACS Applied Materials & Interfaces* **2017**, *9*, 12436.

[7] S. Ma, Y. Wang, C. Fu, Y. Ma, Y. Gao, G. Yin, P. Zuo, *Chemical Communications* **2020**, *56*, 12797.

[8] A. L. Páez Jerez, D. M. Chemes, E. L. Sham, L. E. Davies, A. Y. Tesio, V. Flexer, *ChemistrySelect* **2020**, *5*, 5465.

[9] Z.-Q. Jin, Y.-G. Liu, W.-K. Wang, A.-B. Wang, B.-W. Hu, M. Shen, T. Gao, P.-C. Zhao, Y.-S. Yang, *Energy Storage Mater* **2018**, *14*, 272.

[10] W. Wang, Z. Cao, G. A. Elia, Y. Wu, W. Wahyudi, E. Abou-Hamad, A. H. Emwas, L. Cavallo, L. J. Li, J. Ming, *ACS Energy Letters* **2018**, *3*, 2899.

[11] G. Li, S. Xu, B. Li, T. Xia, J. Yu, F. Shao, H. Li, Z. Yang, Y. Su, Y. Zhang, J. Ma, N. Hu, *ChemElectroChem* **2021**, *8*, 873.

[12] T. Boenke, P. Härtel, S. Dörfler, T. Abendroth, F. Schwotzer, H. Althues, S. Kaskel, *Batteries & Supercaps* **2021**, batt. 202100033.

[13] F. Liu, S. Chiluwal, A. S. Childress, C. Etteh, K. Miller, M. Washington, A. M. Rao, R. Podila, *ACS Applied Nano Materials* **2021**, *4*, DOI 10.1021/acsanm.0c02073.
[14] H. Li, X. Wen, F. Shao, C. Zhou, Y. Zhang, N. Hu, H. Wei, *Chemical Engineering Journal* **2021**, *412*, 128562.
[15] L. Wang, Y.-H. Song, B.-H. Zhang, Y.-T. Liu, Z.-Y. Wang, G.-R. Li, S. Liu, X.-P. Gao, *ACS Applied Materials & Interfaces* **2020**, *12*, 5909.
[16] J. Tan, D. Li, Y. Liu, P. Zhang, Z. Qu, Y. Yan, H. Hu, H. Cheng, J. Zhang, M. Dong, C. Wang, J. Fan, Z. Li, Z. Guo, M. Liu, *Journal of Materials Chemistry A* **2020**, *8*, 7980.
[17] Z. Cui, C. Zu, W. Zhou, A. Manthiram, J. B. Goodenough, *Advanced Materials* **2016**, *28*, 6926.
[18] M. Li, R. Carter, A. Douglas, L. Oakes, C. L. Pint, *ACS Nano* **2017**, *11*, 4877.
[19] R. Yan, M. Oschatz, F. Wu, *Carbon* **2020**, *161*, 162.
[20] Y. Zhou, H. Shu, Y. Zhou, T. Sun, M. Han, Y. Chen, M. Chen, Z. Chen, X. Yang, X. Wang, *Journal of Power Sources* **2020**, *453*, 227896.
[21] H. Chen, W.-D. Dong, F.-J. Xia, Y.-J. Zhang, M. Yan, J.-P. Song, W. Zou, Y. Liu, Z.-Y. Hu, J. Liu, Y. Li, H.-E. Wang, L.-H. Chen, B.-L. Su, *Chemical Engineering Journal* **2020**, *381*, 122746.
